# Reduction-Responsive Cationic Vesicles from Bolaamphiphiles with Ionizable Amino Acid or Dipeptide Polar Heads

**DOI:** 10.1021/acs.langmuir.3c01294

**Published:** 2023-09-20

**Authors:** Ana M. Bernal-Martínez, César A. Angulo-Pachón, Francisco Galindo, Juan F. Miravet

**Affiliations:** Department of Inorganic and Organic Chemistry, Universitat Jaume I, 12071 Castelló de la Plana, Spain

## Abstract

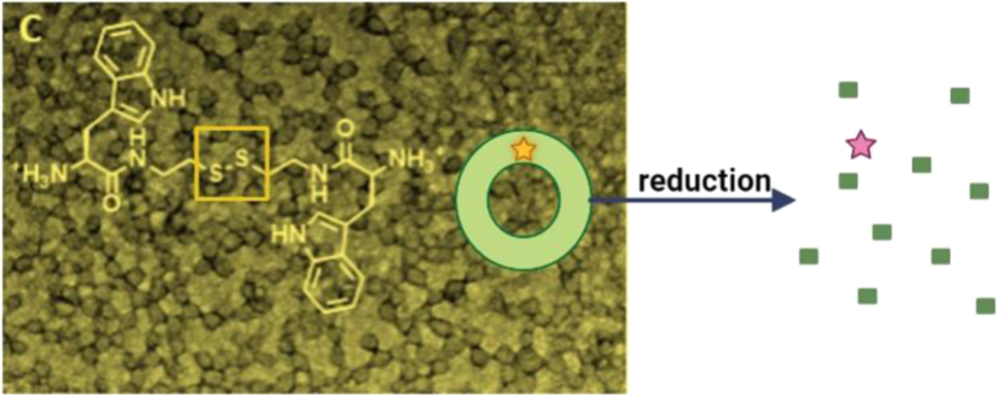

This paper presents a study of the aggregation of cationic bolaamphiphilic molecules into vesicles. These molecules are based on a cystamine core with protonated terminal dipeptide groups. The study found that vesicles can be formed at pH 4 for all of the dipeptide-terminated bolaamphiphiles containing different combinations of l-valine, l-phenylalanine, and l-tryptophan. The concentration for aggregation onset was determined by using pyrene as a fluorescent probe or light dispersion for compounds with tryptophan. Dynamic light scattering (DLS) studies and transmission electron microscopy (TEM) reveal that the vesicles have diameters ranging from 140 to 500 nm and show the capability of loading hydrophobic cargos, such as Nile red, and their liberation in reductive environments. Furthermore, the bolaamphiphiles are only fully protonated and prone to vesicle formation at acidic pH, making them a promising alternative for gastrointestinal delivery.

## Introduction

Cationic vesicles and liposomes have received much attention mainly because of their use as vectors of nucleic acids.^1^ Additionally, cationic vesicles are of therapeutic value because they present affinity for negatively charged surfaces such as those of bacteria,^2^ cells,^3^ or yeasts.^4^

A common approach to obtain cationic liposomes, already reported in the 1980s, is to add a cationic lipid analogue such as 1,2-bis(oleoyloxy)-3-(trimethylammonium)propane (DOTAP) to conventional liposomes formed by natural zwitterionic phospholipids.^5^ Alternatively, in many cases, different cationic gemini surfactants have been used to prepare liposomes when mixed with phospholipids.^6,7^ Another approach involved the synthetic modification of phosphatidylethanolamine with an l-lysine derivative.^8^ Monocomponent cationic vesicles can be formed with compounds that are not based in phospholipids such as deaqualinum, a bolaamphiphile with two terminal quinolinium units,^9,10^ diC14-amidine,^11^ dioctadecyldimethylammonium bromide,^12^ derivatives of vernonia oil,^13^ N-[3-(dimethylamino)propyl]-octadecanamide,^14^ a polypeptide grafted with a polycation,^15^ or gemini surfactants.^16,17^ Also, quaternary ammonium surfactants^18−20^ and amino acid-derived surfactants^21−23^ sometimes evolve into vesicles in the presence of different species.

Despite the variety of cationic vesicles described, there is room for innovation considering aspects such as lowering toxicity, introducing stimuli-responsiveness, and preparing single-component vesicles from inexpensive and easily synthesized compounds. As far as toxicity is concerned, this has been an important issue both in cell cultures and in vivo experiments and constitutes a severe drawback for moving into clinical applications of cationic liposomes.^24−26^

The preparation of stimuli-responsive conventional liposomes has been extensively studied,^27^ but only relatively few cases of stimuli-responsive cationic vesicles have been reported. Cationic vesicles with pH responsiveness were obtained using surfactants with amine groups.^28,29^ Additionally, cationic vesicle formation/disassembly has been controlled by introducing redox responsive units in their structure based on ferrocene,^30,31^ disulfide^17,32,33^, or sulfonamide.^34^ In particular, disulfide units have been employed extensively in a broad class of nanocarriers that release their load upon reaction with glutathione.^35,36^ As for cationic vesicles with disulfide units, it has been reported an increased release of siRNA from lysosomes to the cytoplasm following the cleavage of the disulfide bridge present in the vesicle-forming molecules.^33^ In another case, the redox sensitivity of the disulfide units present in cationic liposomes correlated well with higher plasmid transfection activity.^32^

Recently, much interest has been devoted to ionizable cationic lipids in the formulation of nanocarriers. In gene transfer, the most promising systems contain ionizable cationic lipids with p*K*_a_ values below 7 that exhibit little positive charge at neutral pH, enabling long circulation times and reduced toxicity.^37,38^ Furthermore, this type of lipid increases its ionization degree in the endosomes (pH ca. 5), facilitating the escape of the actives into the cytoplasm, a critical step for the effective transfection.^39^

Bolaamphiphilic molecules are formed by a hydrophobic moiety, for example, an alkyl chain, and two polar hydrophilic units at terminal positions. This type of molecule presents a rich self-assembly behavior in water. Vesicles can be formed with a monolayer lipid membrane, in contrast to bilayer lipid membranes formed in conventional liposomes, which impart improved thermal stability.^40^ There is a relatively low number of bolaamphiphilic cationic vesicles in the literature which present different polar terminal groups such as bis(paraquat),^41^ trimethylammonium,^42−44^ dequalinium,^9^ and quinolinium.^45^

**Scheme 1:**
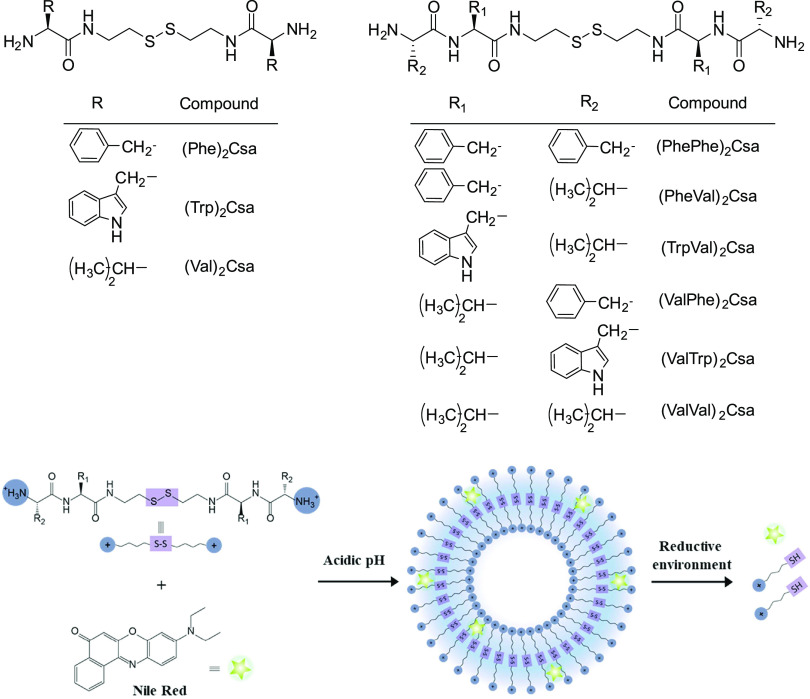
Structure of the Studied Bolaamphiphiles and Pictorial Representation of Vesicle Formation and Reduction Triggered Disassembly

Here we report on new bolaamphiphiles (Scheme 1) with terminal ionizable amino groups containing a reduction-sensitive disulfide moiety and their aggregation into stimuli-responsive cationic vesicles. Additionally, the incorporation and release of the dye Nile red, as a model hydrophobic compound, in the vesicles are studied (Scheme 1).

## Experimental Section

### Materials

Cystamine dihydrochloride (97%), *N*,*N*-diisopropylethylamine (DIPEA, peptide synthesis grade), *N*-carbobenzyloxy-l-valine (>99%), *O*-(benzotriazol-1-yl)-*N*,*N*,*N*′,*N*′-tetramethyluronium tetrafluoroborate (TBTU, 99%), *N*-carbobenzyloxy-l-tryptophan (98%), hydrogen bromide (pure, 33 wt % solution in glacial acetic acid), and Nile red (99%) were purchased from Thermo Fisher Scientific. 1,8-Diazabicyclo[5.4.0]undec-7-ene (DBU, ≥99.0%), sodium hydroxide pellets (≥98.0%), and pyrene (98%) were ordered from Sigma-Aldrich. *N*-Carbobenzyloxy-l-phenylalanine (99.6%) was ordered from Cymitquimica-Bachem; hydrochloric acid (37%) from LABBOX-España; magnesium sulfate anhydrous, extrapure, from Scharlau; uranyl acetate 1% solution (depleted uranium) from Electron Microscopy Sciences; and tris(2-carboxyethyl)phosphine hydrochloride (TCEP, >98%) from TCI chemicals. Acetate buffer was prepared by dissolving sodium acetate anhydrous (99%) and glacial acetic acid (99%) in water, both purchased from Thermo Fisher Scientific.

### Equipment

^1^H/^13^C NMR spectra were recorded at 400/101 MHz or 300/75 MHz in the indicated solvent at 30 °C. Signals of the deuterated solvent (DMSO-*d*_6_ in all cases, unless otherwise indicated) were taken as the reference in DMSO-*d*_6_, i.e., the singlet at δ 2.50 and the quadruplet centered at 39.52 ppm for ^1^H and ^13^C NMR, respectively. ^1^H and ^13^C signals were assigned with the aid of 2D methods (COSY, HSQC, and HMBC).

The mass spectra were run in electrospray ionization mode (ESI-MS). Mass spectra were recorded on a mass spectrometry triple Quadrupole Q-TOF Premier (Waters) with simultaneously equipped with electrospray and APCI probes.

The absorbance and fluorescence properties were measured with a JASCO V-630 UV–vis spectrophotometer and a JASCO FP-8300 fluorometer, both equipped with a Peltier accessory and measured at 25 °C.

DLS size distribution and ζ-potential measurements were recorded using a Zetasizer Nano-ZS90 instrument (Malvern Instruments, U.K.).

Transmission electron microscopy (TEM) images were taken on a JEM-2100 Plus microscope with a LaB6 200 kV thermionic gun LaB6 200 kV, equipped with a high-resolution camera and a CMOS sensor.

### General Synthetic Procedure

All of the compounds were synthesized following standard peptide chemistry, as shown in Scheme S1. See details in the Supporting Information.

### Determination of p*K*_a_

Potentiometric titrations to determine acid–base constants were carried out at 298 K. At acidic pH, the compounds were found to be very soluble in water, with solubilities higher than 25 mg mL^–1^ in all cases (the solubility limit was not determined). In a typical experiment, 7 mL of a solution of the corresponding compound (15 mg) in HCl (0.1 M) was titrated with standardized 0.1 M NaOH under stirring. Next, the base was added with a NE-300 Just Infusion Syringe Pump (0.04 mL min^–1^, inner diameter 14.57 mm) using an SGE Analytical Science syringe (10 mL), which had a connected needle of stainless steel (Luer Look, 0.7 mm × 300 mm). The pH was monitored every 10 s (S220 Seven Compact pH-meter, Mettler Toledo). The p*K*_a_ values were calculated by fitting the experimental data to calculated titration curves with the program HYPERQUAD.

The ionization degree was calculated from the p*K*_a_ values by using the speciation program HYSS.

### Critical Aggregation Concentration

The critical aggregation concentration was determined by fluorescence using pyrene as a probe (peak I/peak III ratio). Different concentrations of the compounds were prepared (0–20 mg mL^–1^) by dissolving the corresponding compound in a solution of 1 μM pyrene in acetate buffer (0.1 M, pH 4, filtered through a cellulose acetate 0.45 μm mesh filter). Although the compounds are fully soluble, resulting in clear solutions, the system was ultrasonicated for approximately 10 min. This follows a standard procedure in our laboratories that was used previously for poorly water-soluble compounds. Pyrene solutions were excited at 334 nm, and the emission spectrum was collected from 350 to 500 nm.

For the critical aggregation concentration determined by turbidimetry, different samples were prepared (0–20 mg mL^–1^) by dissolving the corresponding compound in acetate buffer (0.1 M, pH 4, filtered through a 0.45 μm mesh filter). The system was ultrasonicated for ca. 10 min. The changes in the absorbance signal at 800 nm were measured.

### Dynamic Light Scattering

Automatic optimization of beam focusing and attenuation was applied to each sample. Samples were introduced into 3 mL disposable PMMA cuvettes (10 mm optical path length). The particle size was reported as the average of three measurements at 25 °C.

### ζ-Potential

ζ-potential measurements were performed at 25 °C by Laser Doppler Microelectrophoresis. The 1 mL sample was taken in disposable folded capillary zeta cells (Malvern, DTS1070).

### Transmission Electron Microscopy

The corresponding solution was deposited directly onto 200-mesh Formvar/Carbon supported copper grids (FCF-200 Cu) for 2 min. Then, the liquid was carefully removed by capillary action by using a filter paper. The grids were immediately stained with one drop of 1% uranyl acetate for 1 min. The excess stain was removed by capillarity. The grids were air-dried before observation.

### ^1^H NMR Study of the Reduction with TCEP

For the NMR study of reduction triggered by TCEP, (ValPhe)_2_Csa (18.5 mmol) was dissolved in 1.85 mL of acetate buffer (0.1 M, pH 4, filtered through a cellulose acetate 0.45 μm mesh filter). The system was ultrasonicated for ca. 10 min. Then, 0.15 mL of TCEP (160 mM, in 0.1 M acetate buffer, pH 4) was added and mixed (vortex) to obtain a solution that was 9.3 mM in (ValPhe)_2_Csa and 12 mM in TCEP. After 16 h, the sample was lyophilized and dissolved in DMSO-d_6_.

### Entrapment and Release of Nile Red

Emission and absorption were recorded before and after 16 h of mixing each compound (5 mM) with TCEP (14 mM). Initially, 1 mL of a stock solution of each compound (10 mM in MeOH) was placed in a cylindrical glass vial. Then, 20 μL of a stock solution of Nile red (1 mM in EtOH) was added. The mixture was evaporated to dryness, followed by the addition of an acetate buffer (0.2 mL, 0.1 M, pH 4). The system was ultrasonicated for 10 min. Then, 0.2 mL of TCEP (160 mM, in 0.1 M acetate buffer pH 4) was added, and the corresponding measurements were carried out after 16 h. The emission spectra were recorded at different excitation wavelengths for each compound, ranging from 555 to 586 nm.

## Results and Discussion

Nine bolaamphiphilic compounds containing a central unit derived from cystamine and a terminal unit corresponding to amino acids (three compounds) or a dipeptide (six compounds) were prepared. l-Phenylalanine, l-valine, and l-tryptophan were used as building blocks (Scheme 1). These amino acids were chosen because they present nonionizable and hydrophobic side chains, aiming to promote aggregation in an aqueous medium. The preparation of the bolaamphiphiles was straightforward, starting from commercially available cystamine and attaching the amino acids with the conventional synthetic methodology used in peptide chemistry (details in the Experimental Section). Some of the compounds described here and related ones were prepared recently in a study of coacervation in a neutral medium.^46^

### Acid–Base Properties

An important characteristic of the bolaamphiphiles studied in this work is the pH-dependent ionizable nature of the amino-terminal groups. Accordingly, the p*K*_a_ determination of the different compounds was carried out by potentiometric titration. The results are presented in Table 1. Although the p*K*_a_ value of the protonated amines is ca. 10, the values in Table 1 range approximately from 6 to 8. This notable p*K*_a_ shift is associated with the hydrophobic and aggregation-prone nature of the molecules. Therefore, the p*K*_a_ values obtained could be considered apparent constants that reflect the thermodynamics of the protonation process and the aggregation taking place upon charge neutralization. This behavior has been reported previously, for example, in the study of hydrogel formation upon neutralization of carboxylic acids.^47,48^ The compounds with lower p*K*_a_ values (p*K*_a_ < 7) correspond to derivatives containing the amino acids phenylalanine and tryptophan, which contain hydrophobic and aromatic side chains. The calculated degree of ionization (α in Table 1) of the bolaamphiphilic diamines at pH 7 ranges from 0.1 for (PhePhe)_2_Csa to 0.47 for (ValVal)_2_Csa. Therefore, these molecules are only partially protonated at neutral pH and present different degrees of protonation. As a result, most of the compounds were only partially soluble at pH 7 because of the substantial presence of neutral species. Consequently, cationic vesicle formation was studied for fully protonated compounds at pH 4. Although interesting, given the complexity introduced by the partial protonation mentioned, the study of aggregation at neutral pH and the variation of vesicle properties and stability with pH is left for a future report.

**Table 1:** Acidity Constants, Ionization Degree, and Critical Aggregation Concentration (CAC) at pH 7 of the Studied Compounds

compound	p*K*_a_1	p*K*_a_2	α (pH 7)^a^	CAC (mM)
(Val)_2_Csa	7.5	6.7	0.38	2.4
(Trp)_2_Csa	6.7	5.9	0.17	1.4
(Phe)_2_Csa	7.9	7.1	0.44	5.5
(ValVal)_2_Csa	8.2	7.6	0.47	1.6
(TrpVal)_2_Csa	7.1	6.0	0.28	0.7
(ValTrp)_2_Csa	6.3	6.5	0.08	1.0
(PheVal)_2_Csa	7.5	6.7	0.38	2.5
(ValPhe)_2_Csa	7.8	7.5	0.43	0.6
(PhePhe)_2_Csa	6.4	6.6	0.10	1.9

a Ionization degree, namely, the ratio of protonated amino groups and total amino groups.

### Determination of the Critical Aggregation Concentration

**Figure 1:**
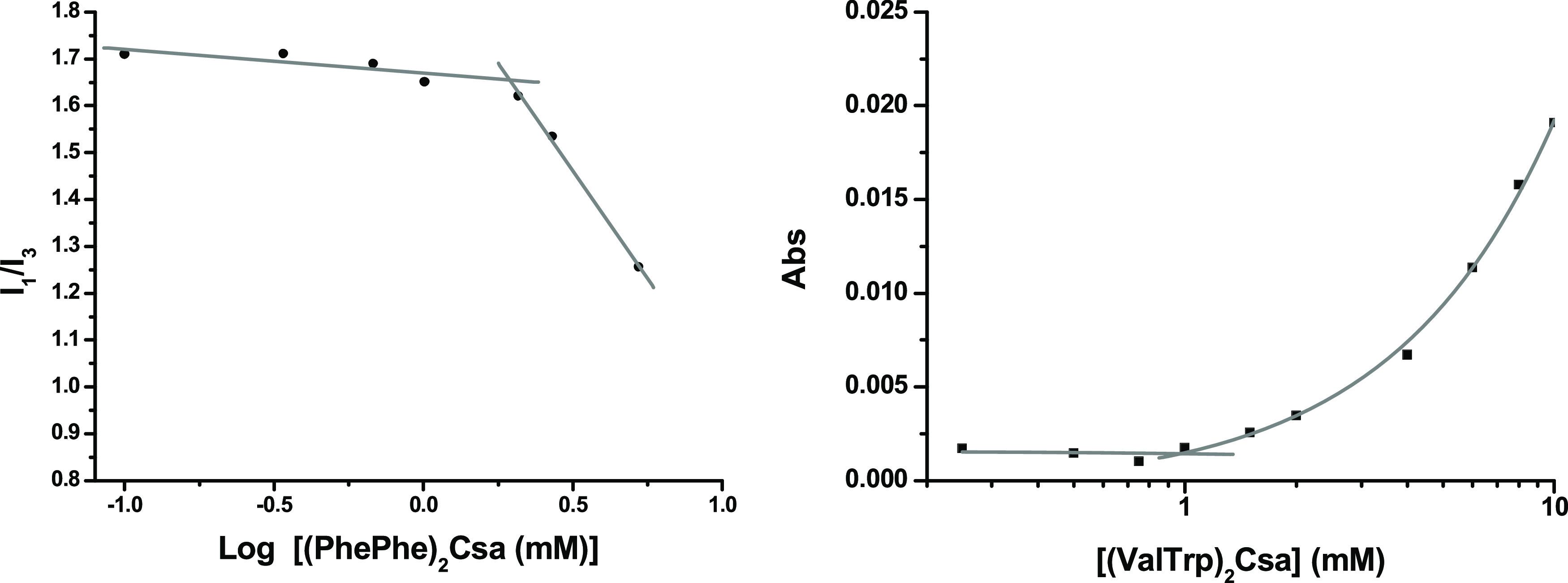
Determination of the critical aggregation concentration using pyrene fluorescence (left) and UV–vis absorption at 800 nm (right).

Pyrene was employed as a fluorescent probe to determine the critical aggregation concentration (CAC) of the bolaamphiphiles at pH 4 (acetate buffer, 0.1 M). It has been shown extensively that the ratio of the first (371 nm) and third (382 nm) peaks of the emission spectrum of pyrene is susceptible to the polarity of the environment and is especially suited to detect the formation of micelles or vesicles.^49^ An example is shown in Figure 1, left (see the other plots in Figure S3). The ratio *I*_I_/*I*_III_ varies drastically at a concentration of (Phe)_2_Csa of 0.2 mM, indicating the onset of aggregation. This methodology was used to obtain CAC values of all of the compounds except those containing a tryptophan unit. The fluorescence of the indole unit of tryptophan precludes proper determination of CAC by pyrene emission studies; hence, turbidometry studies were performed for this purpose. The light dispersed at 800 nm, measured with a UV–vis spectrometer, was plotted versus the concentration of the studied compound. A representative example is shown for compound (ValTrp)_2_Csa in Figure 1, right (see the other plots in Figure S4). The baseline of the UV–vis spectrum remains unchanged until an exponential increase occurs at a concentration of about 1 mM, indicating the formation of aggregated species that disperse the light. The CAC values obtained range from 0.7 to 2.5 mM except for the case of (Phe)_2_Csa, with a value of 5.5 mM (see Table 1).

### Vesicle Study by DLS and TEM

**Figure 2:**
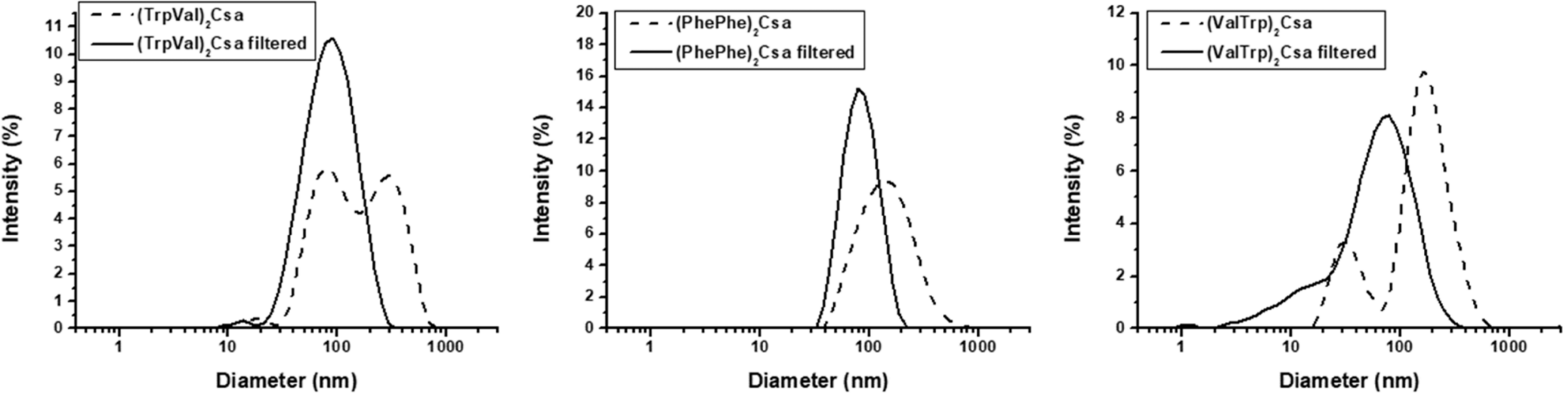
Size distribution graphs obtained by DLS for 6 mM samples, pH = 4.

Vesicle formation at pH 4 was studied in the first instance by dynamic light scattering (DLS). For this purpose, the particle size distribution of 6 mM samples was obtained (Table 2). Good correlograms were obtained (Figure S1), indicating the presence of particles with intensity-averaged diameters in the range 140–230 nm, except for the case of (ValVal)_2_Csa with *D*_i_ = 526 nm, therefore supporting vesicle formation. Some of the size distribution graphs are shown in Figure 2; the rest can be found in Figure S1. The samples showed moderate to high polydispersity with PdI values ranging from 0.28 to 0.65 (a higher PdI value indicates a higher polydispersity, with the maximum possible value 1). Compounds (PhePhe)_2_Csa, (PheVal)_2_Csa, and (ValTrp)_2_Csa show a monomodal size distribution being bimodal for the other ones. The capability of forming vesicles is maintained in all seven compounds shown in Table 1, despite the different amino acid units present.

**Table 2:** DLS Analysis of the Vesicles Formed at pH = 4 for Samples with a Concentration of 6 mM^a^

compound	*D*_i_ (nm)	*D*_n_ (nm)	PdI	count rate^b^ (kcps × 10^–3^)	ζ-potential (mV)
(PhePhe)_2_Csa	150 ± 16	77 ± 29	0.28 ± 0.08	50.6 ± 0.3	55 ± 2
(PheVal)_2_Csa	212 ± 31	64 ± 16	0.51 ± 0.08	3.5 ± 0.2	36 ± 3
(Trp)_2_Csa	252 ± 5^c^	63 ± 2	0.53 ± 0.01	3.9 ± 0.1	35 ± 4
(TrpVal)_2_Csa	230 ± 31^c^	61 ± 15	0.44 ± 0.1	59.1 ± 3	55 ± 2
(ValPhe)_2_Csa	222 ± 21^c^	116 ± 41	0.46 ± 0.02	4.1 ± 0.2	40 ± 3
(ValTrp)_2_Csa	279 ± 4	61 ± 5	0.46 ± 0.03	9.2 ± 0.3	38 ± 2
(ValVal)_2_Csa	557 ± 99^c^	378 ± 98^c^	0.24 ± 0.04	6.9 ± 0.3	29 ± 4

a *D*_i_: Diameter obtained from the intensity distribution; *D*_n_: number-averaged diameter distribution; and PdI: polydispersity index

b Derived count rate (representative of the scattering intensity that would be measured in the absence of the laser attenuation filter).

c Bimodal distribution.

However, the nature of the amino acids and whether they are present as a single amino acid or a dipeptide significantly influence the formation of vesicles. Table 2 does not include two compounds with a single amino acid building block, namely, (Val)_2_Csa and (Phe)_2_Csa, which exhibited count rates below 1000 kcps. This indicates a low concentration of vesicles. It is reasonable to assume that the critical aggregation concentration observed for these molecules corresponds to the micelle formation process. Micelles generate significantly less light dispersion compared to larger objects such as vesicles, making them undetectable by DLS at the concentrations examined in this study.

On the other hand, (Trp)_2_Csa showed better DLS data with a higher count rate and good correlograms. The extended aromatic indole unit in tryptophan and its hydrophobic supramolecular interactions could explain this difference. All of the compounds containing a dipeptide block showed significant count rates and good DLS correlograms. It seems that the additional amino acid unit considerably favors the aggregation, a fact that could be ascribed to the higher hydrophobic nature of these molecules. For example, the value of *C* log *P* (fragment-based computation of the partition coefficient between water and octanol, obtained using ChemDraw software) is 2.0 for (Phe)_2_Csa but for the dipeptide derivative (PhePhe)_2_Csa is notably higher with a value of 4.3. The intensity of scattered light samples of (PhePhe)_2_Csa and (TrpVal)_2_Csa is about 1 order of magnitude higher than the other compounds, indicating an elevated concentration of vesicles. In the case of (PhePhe)_2_Csa, it has to be recalled that the dipeptide PhePhe and its derivatives show a high tendency to form aggregated species in water.^50^ To evaluate the surface charge of the vesicles, the ζ-potential was determined by electrophoretic mobility using the DLS equipment. A positive potential was obtained for all of the vesicles with values between 29 and 55 mV, associated with good colloidal stability (see phase plots in Figures S2 and S3).^51^

The samples of vesicles were passed through a 200 nm cellulose acetate filter to obtain a monomodal and narrower size distribution and remove large aggregates. This procedure proved to be successful for compounds (PhePhe)_2_Csa, (TrpVal)_2_Csa, and (ValTrp)_2_Csa (see Figure 2 and Table 3), affording monodisperse and narrow size distribution with *D*_i_ values ranging from 66 to 85 nm. The particle size was notably reduced after extrusion, which is a commonly observed characteristic in vesicles.^52^ However, the samples of the other compounds exhibited low count rates (below 1000 kcps), indicating that most of the vesicles were trapped in the filter.

**Table 3:** DLS Analysis of the Vesicles Formed at pH 4 for Samples with a Concentration of 6 mM After Extrusion Through a 200 nm Cellulose Acetate Filter^a^

compound	*D*_i_ (nm)	PdI	count rate (kcps × 10^–3^)	ζ-potential (mV)
(PhePhe)_2_Csa	82 ± 1	0.11 ± 0.02	14.0 ± 0.2	39 ± 4
(TrpVal)_2_Csa	85 ± 1	0.22 ± 0.01	18.3 ± 0.5	30 ± 1
(ValTrp)_2_Csa	66 ± 6	0.56 ± 0.03	2.12 ± 0.02	31 ± 3

a *D*_i_: Diameter obtained from the intensity distribution; PdI: polydispersity index

**Figure 3:**
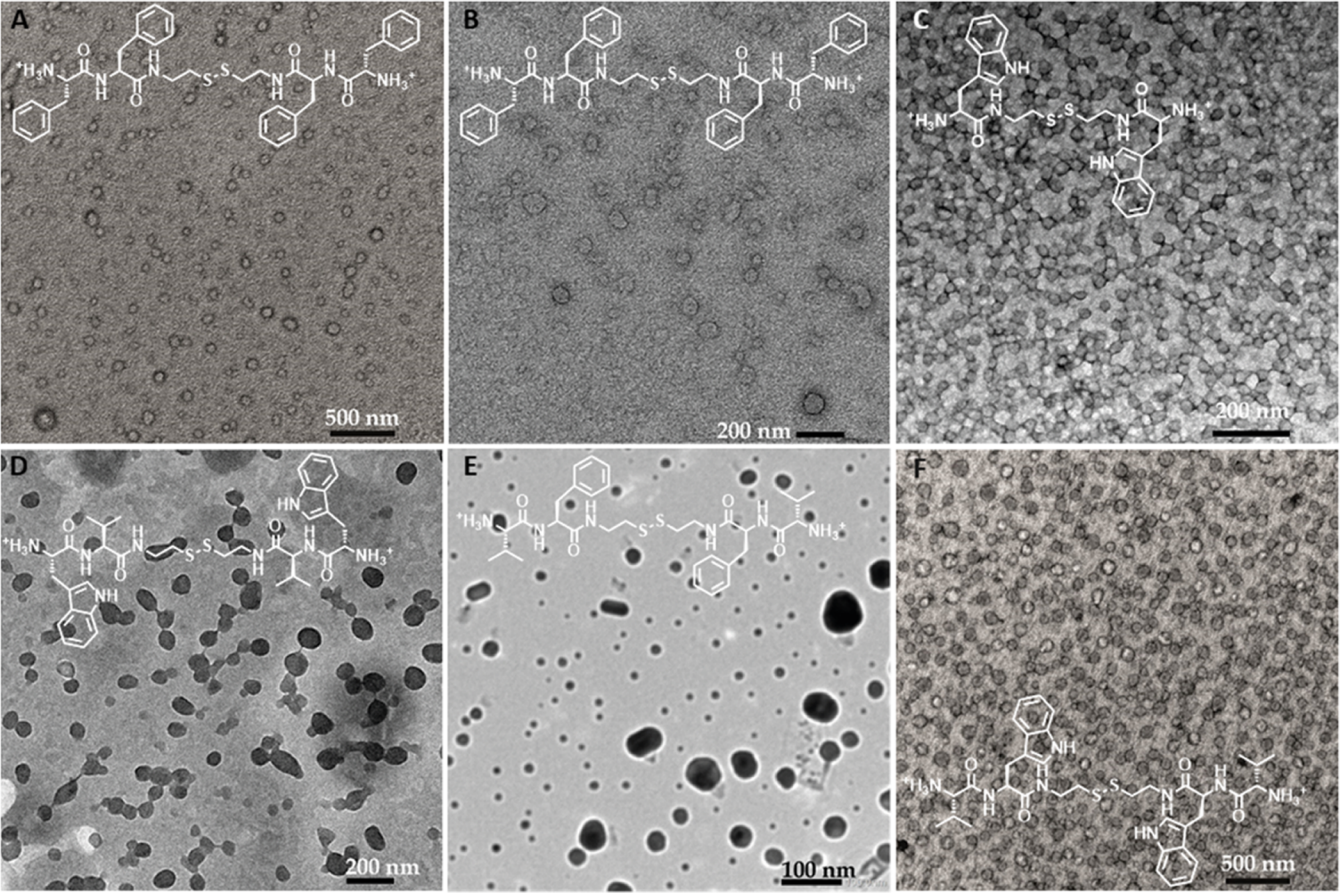
TEM images of the vesicles obtained at pH = 4 (uranyl acetate staining). (A, B) (PhePhe)_2_Csa; (C) (Trp)_2_Csa; (D) (TrpVal)_2_Csa; (E) (ValPhe)_2_Csa; and (F) (ValTrp)_2_Csa.

Vesicle formation could be confirmed for five molecules by transmission electron microscopy (TEM) using uranyl acetate staining (Figure 3). Spherical objects could be observed homogeneously distributed in the sample with rather monodisperse diameters, which were consistent with the values obtained by DLS. It must be noted that although PdI values in Tables 2 and 3 indicate a relatively broad distribution of sizes, those values correspond to intensity-averaged diameters. On the other hand, number-averaged diameters (*D*_n_) present a narrower distribution. In general, the observed objects in TEM micrographs support the formation of vesicles (Figure 3A–C,3 F). In Figure 3D,E, the particles resemble coacervates, which are liquid–liquid-phase-separated droplets mainly composed of water. However, it is important to note that staining effects cannot be ruled out as a contributing factor to the observed appearance. Additionally, in Figure 3E, objects with a diameter smaller than 10 nm coexist with larger aggregates, which can be attributed to micelles. It is worth mentioning that micelles and vesicles often coexist or can undergo transformations into each other.^53^

### Reductive Vesicle Disassembly and Nile Red Loading

The sensitivity of the bolaamphiphiles to reduction was demonstrated by ^1^H NMR. For example, the spectra of (ValPhe)_2_Csa before and after reductive treatment with tris(2-carboxyethyl)phosphine (TCEP)^54^ are shown in Figure S5. An upfield shift of ca. 0.2 ppm of the methylene group vicinal to the sulfur atom is observed upon reduction, as reported previously in related molecules.^55^

**Figure 4:**
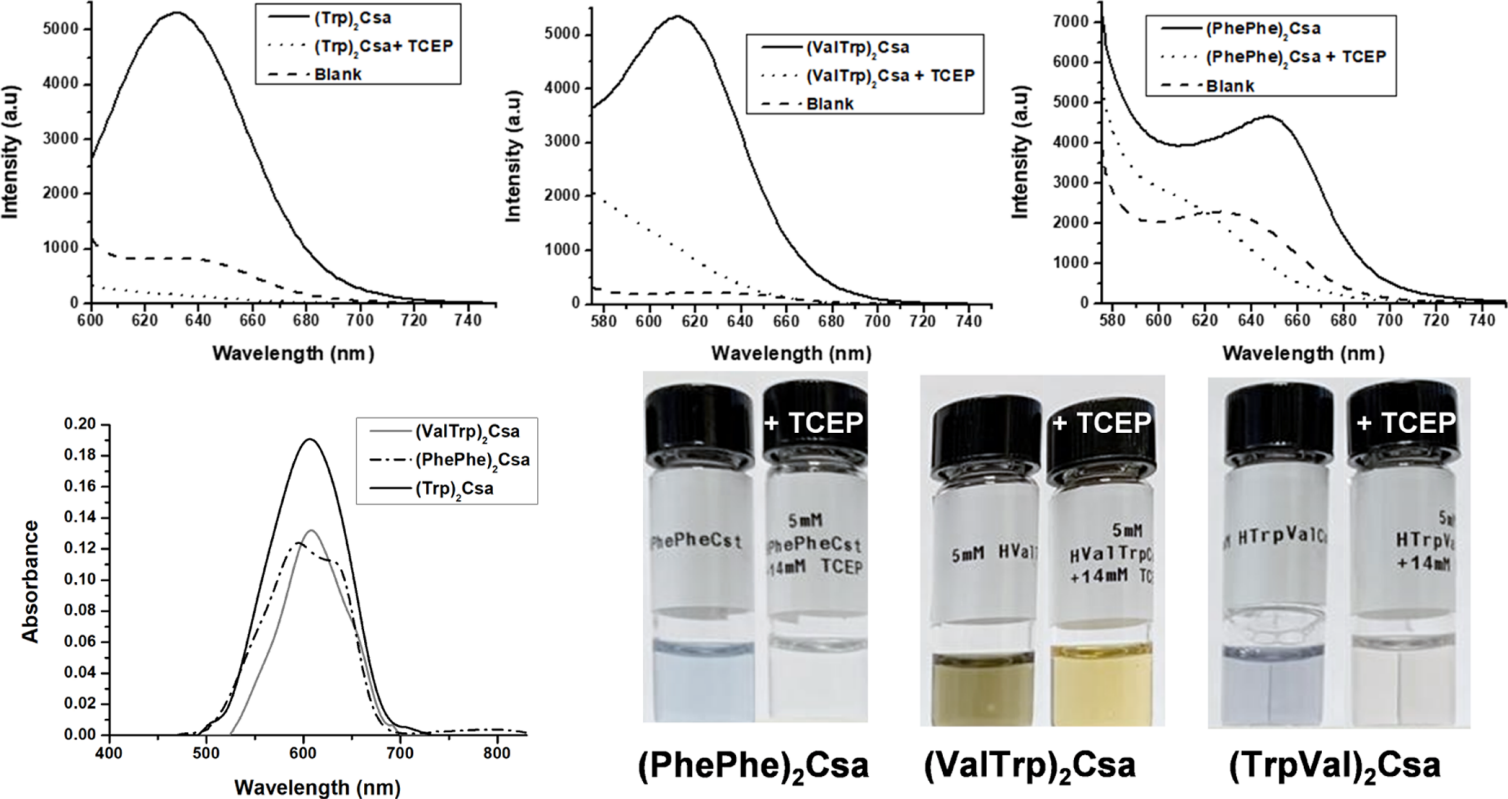
Top: Fluorescence spectra of Nile red (10 μM), pH = 4, in the presence of vesicles and after their reductive treatment with TCEP. (Trp)_2_Csa: λ_exc_ = 586 nm, slitwidths 10–10 mm; (TrpVal)_2_Csa: λ_exc_ = 546 nm, slitwidths 20–10; and (PhePhe)_2_Csa: λ_exc_ = 555 nm, slitwidths 20–10 mm. Bottom: UV–vis spectra and pictures of Nile red samples (10 μM) in the presence of vesicles at pH = 4. Inset: picture of the samples before and after treatment with TCEP.

The interaction between the vesicles and Nile red, a hydrophobic dye that exhibits fluorescence when incorporated into hydrophobic domains,^56^ was studied to assess the potential of the vesicles as carriers for hydrophobic substances. Figure 4 (top) shows the fluorescence spectra obtained for the different vesicles in the presence of 10 mM Nile red and those obtained after reduction using TCEP. In the presence of vesicles, the Nile red fluorescence is activated due to its incorporation in the hydrophobic domains of the vesicles. Reductive disassembly of the vesicles results in a significant reduction of the emission, attributed to the release of Nile red to the aqueous environment (see the study with the rest of the compounds in Figure S6).

The entrapment and release of Nile red can be observed visually using UV–vis spectroscopy. The presence of vesicles leads to bluish-colored solutions that become significantly decolorized upon reduction with TCEP (Figure 4, bottom). It should be noted that Nile Red is a dye with prominent solvatochromic properties, which means it exhibits various colors depending on the polarity of the medium.^57^

## Conclusions

Based on the findings presented above, it is evident that the bolaamphiphilic structure, consisting of a cystamine core with protonated terminal dipeptide groups, serves as a robust motif for the formation of cationic vesicles in an aqueous acidic medium. The incorporation of dipeptide terminal units in the bolaamphiphiles promotes vesicle formation compared to compounds with single amino acid units, which can be attributed to their increased hydrophobic nature. Notably, the presence of aromatic and hydrophobic amino acids such as l-phenylalanine and l-tryptophan facilitates the aggregation process, leading to the formation of cationic vesicles.

Pyrene fluorescence analysis proves to be a convenient method for evaluating the aggregation onset concentrations of these molecules, with the exception of those containing the fluorescent l-tryptophan unit. In such cases, light dispersion measurements offer a suitable alternative procedure.

Furthermore, extrusion through a 200 nm filter has been demonstrated as a successful technique for obtaining vesicles with improved diameter polydispersity.

The sensitivity of the vesicles to reduction is confirmed by ^1^H NMR spectroscopy. Additionally, the use of Nile red as a probe conveniently demonstrates the incorporation of hydrophobic molecules into the vesicles and their responsiveness to reduction.

One important limitation of the reported systems is that the bolaamphiphiles are fully protonated and, therefore, prone to vesicle formation only under acidic conditions. Although the initial design aimed to achieve functionality at physiological pH, a significant aggregation-driven shift in the p*K*_a_ values of the terminal amino units occurs, resulting in a range of p*K*_a_ values from 6.4 to 8.2. Nonetheless, these findings provide insights for the design of new families of bolaamphiphiles with appropriate terminal groups that maintain a positive charge at neutral pH, such as guanidinium or trialkylammonium.

In terms of advantages, these systems demonstrate robustness and stability under acidic conditions, such as those encountered in the gastric environment. Therefore, the bolaamphiphilic vesicles reported herein show potential as carriers for gastrointestinal delivery, offering a promising alternative for pharmaceutical transportation. It is worth noting that liposomes formed by the aggregation of common phospholipids possess ester linkages with low resistance to the acidic gastric pH and enzymatic degradation.^58,59^

## Supporting Information Available

DLS and ζ-potential plots, graphs for the determination of CAC, ^1^H NMR study of the reduction reaction, fluorescence spectra of the study with Nile red, and detailed synthetic procedures and characterization of the compounds (PDF)
