# Supplementary material for: Reduction-Responsive Cationic Vesicles from Bolaamphiphiles with Ionizable Amino Acid or Dipeptide Polar Heads
Source: Langmuir. 2023 Sep 20;39(39):13841–9. doi: 10.1021/acs.langmuir.3c01294 (PMC10552552; doi:10.1021/acs.langmuir.3c01294)
Supplement: Supplementary file 1 — la3c01294_si_001.pdf [file la3c01294_si_001.pdf]

SUPPORTING INFORMATION

Reduction-Responsive Cationic Vesicles from Bolaamphiphiles with Ionizable Amino Acid or Dipeptide Polar Heads

Ana M. Bernal-Martínez, César A. Angulo-Pachón, Francisco Galindo, Juan F. Miravet**

Department of Inorganic and Organic Chemistry

Universitat Jaume I

12071 Castelló de la Plana, Spain

E-mail: francisco.galindo@uji.es, miravet@uji.es

TABLE OF CONTENTS

Topic	page
DLS SIZE DISTRIBUTION GRAPHS	S2
Z-POTENTIAL PHASE PLOTS	S3
ClogP VALUES	S5
CRITICAL AGGREGATION GRAPHS	S6
^{1}H NMR STUDY OF REDUCTION WITH TCEP	S8
FLUORESCENCE SPECTRA	S9
SYNTHESIS AND CHARACTERIZATION	S10

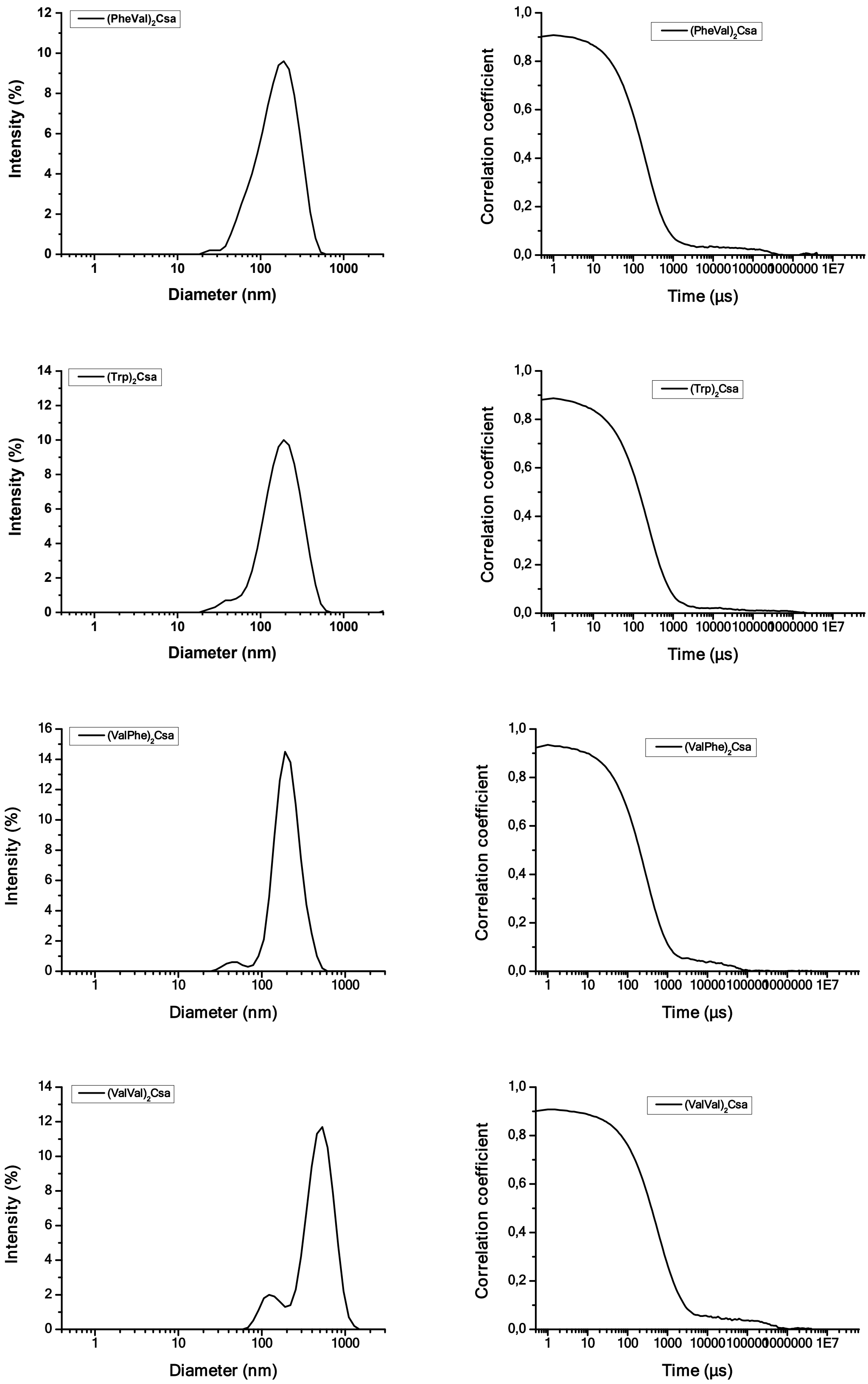

Figure S1. Plots of diameter distribution and correlograms obtained by DLS.

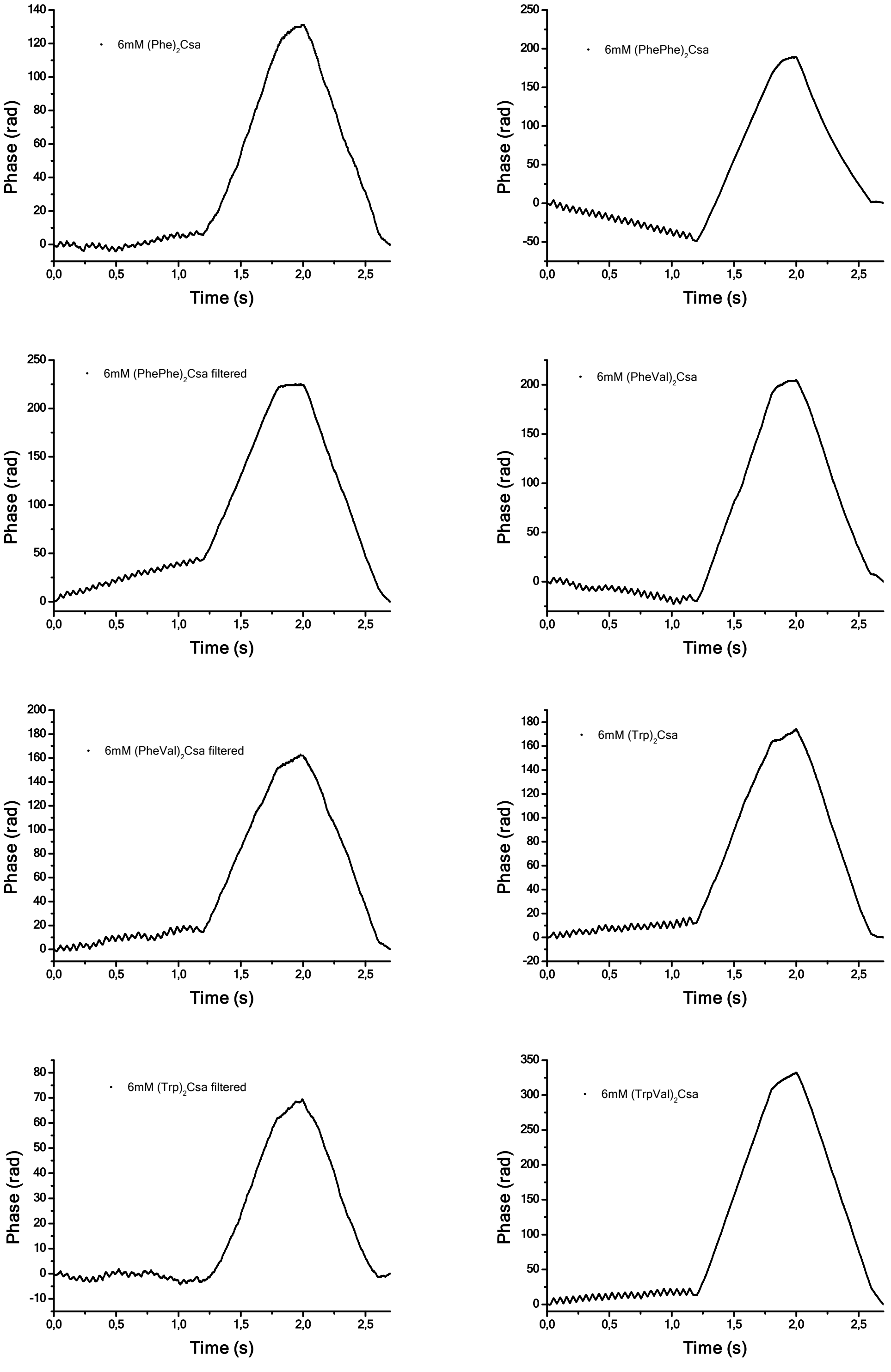

Figure S2. ζ-potential phase plots.

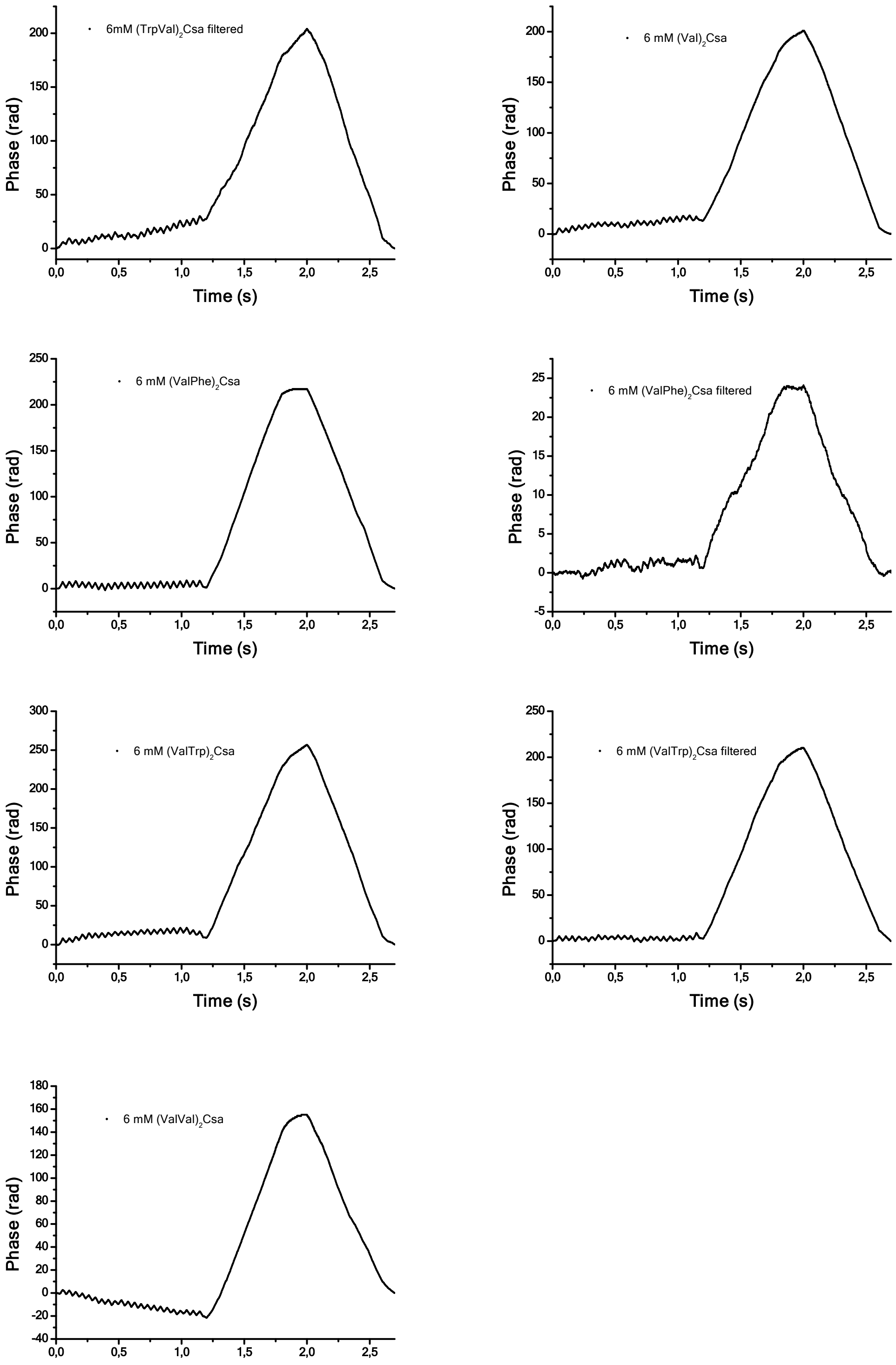

Figure S3. ζ-potential phase plots (I).

Table S1. Partition coefficients between water and octanol using a fragment-based computation obtained with ChemDraw software.

COMPOUND	clogP (calculated)
$(Phe)_2Csa$	2.0
$(PhePhe)_2Csa$	4.3
$(PheVal)_2Csa$	3.3
$(Trp)_2Csa$	1.9
$(TrpVal)_2Csa$	3.3
$(Val)_2Csa$	1.0
$(ValPhe)_2Csa$	3.3
(ValTrp)Csa	3.3
(ValVal)Csa	2.3

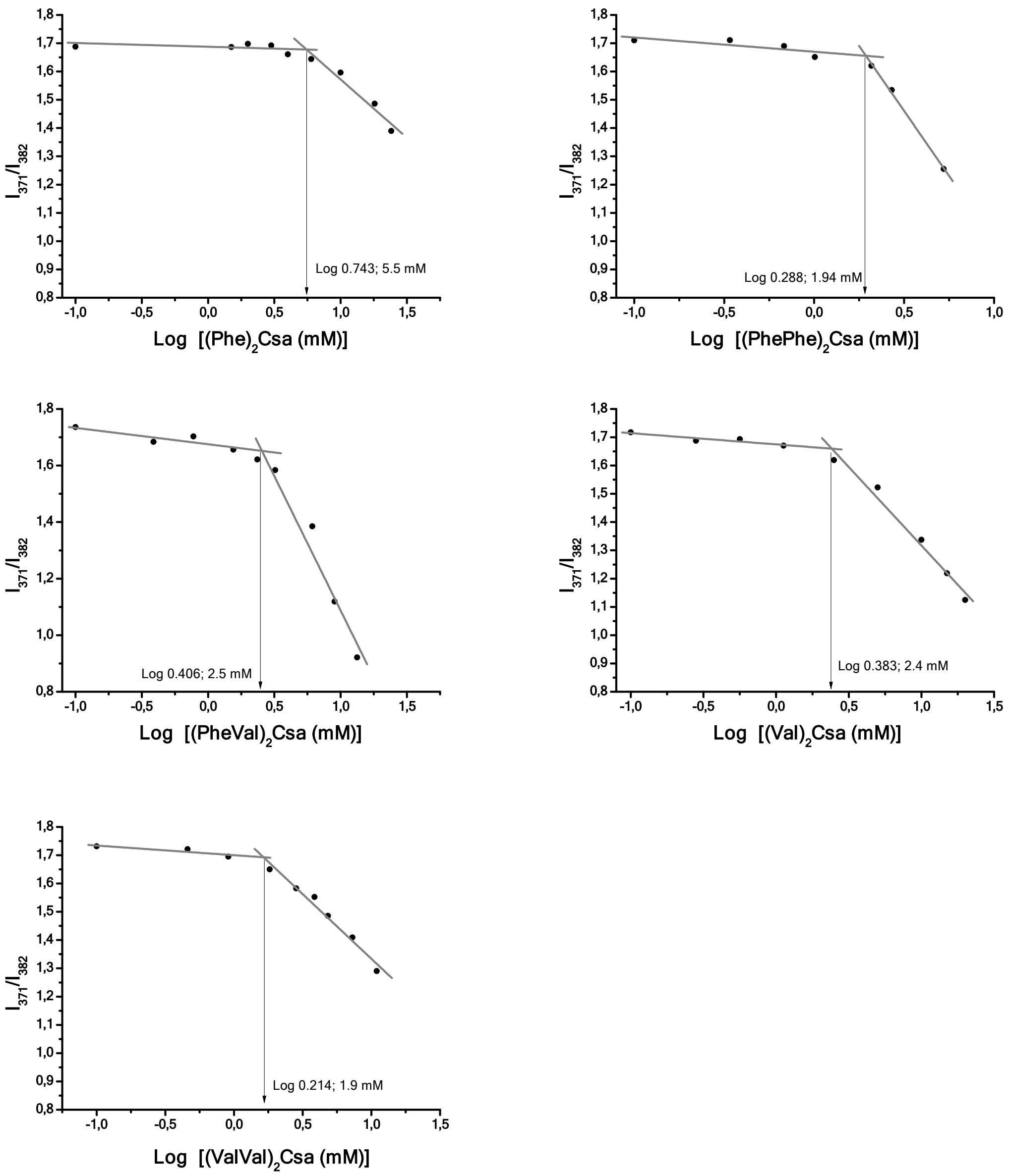

Figure S4. Determination of the critical aggregation concentration (CAC) using pyrene as a fluorescent probe.

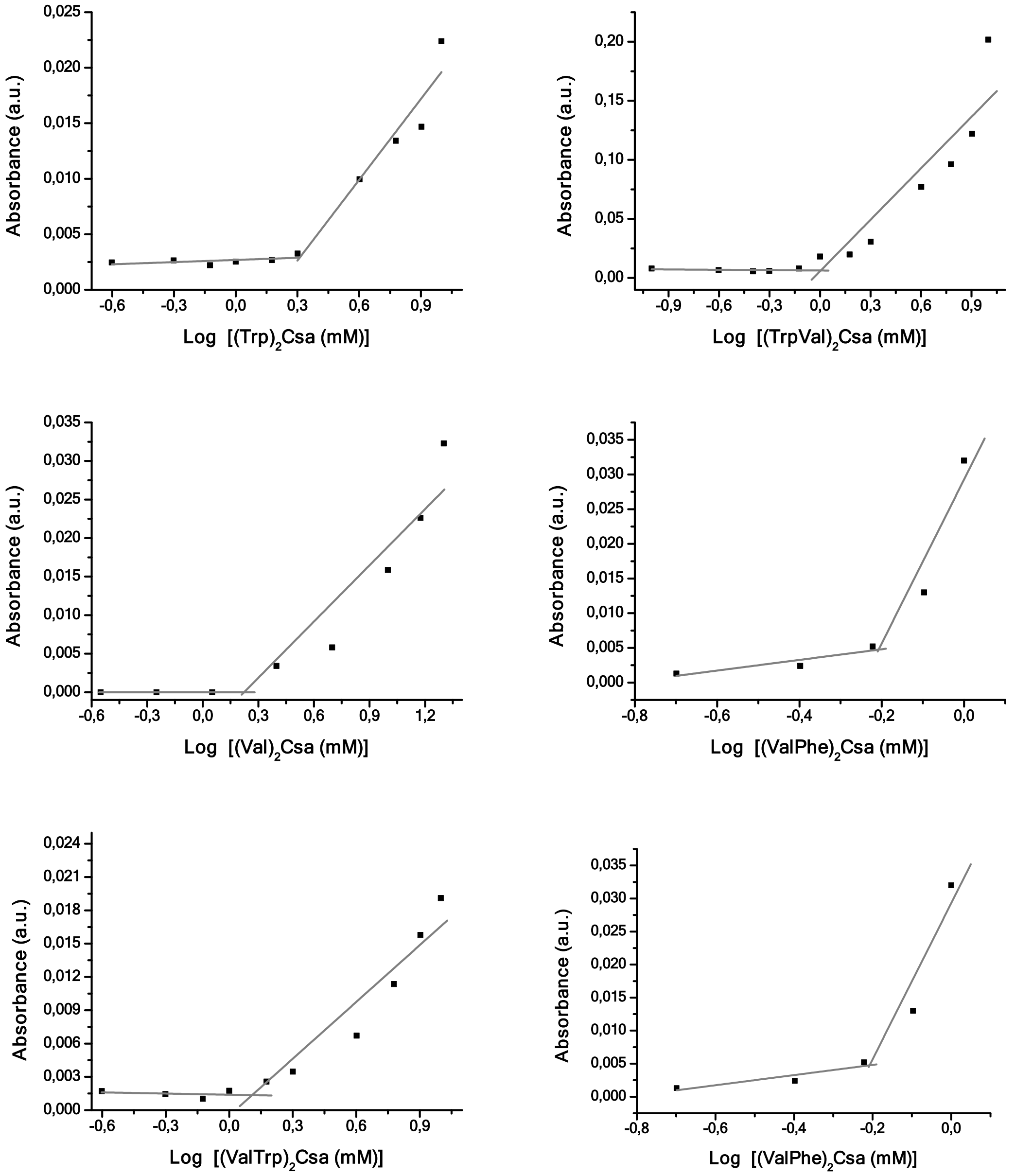

Figure S5. Determination of the critical aggregation concentration by turbidimetry.

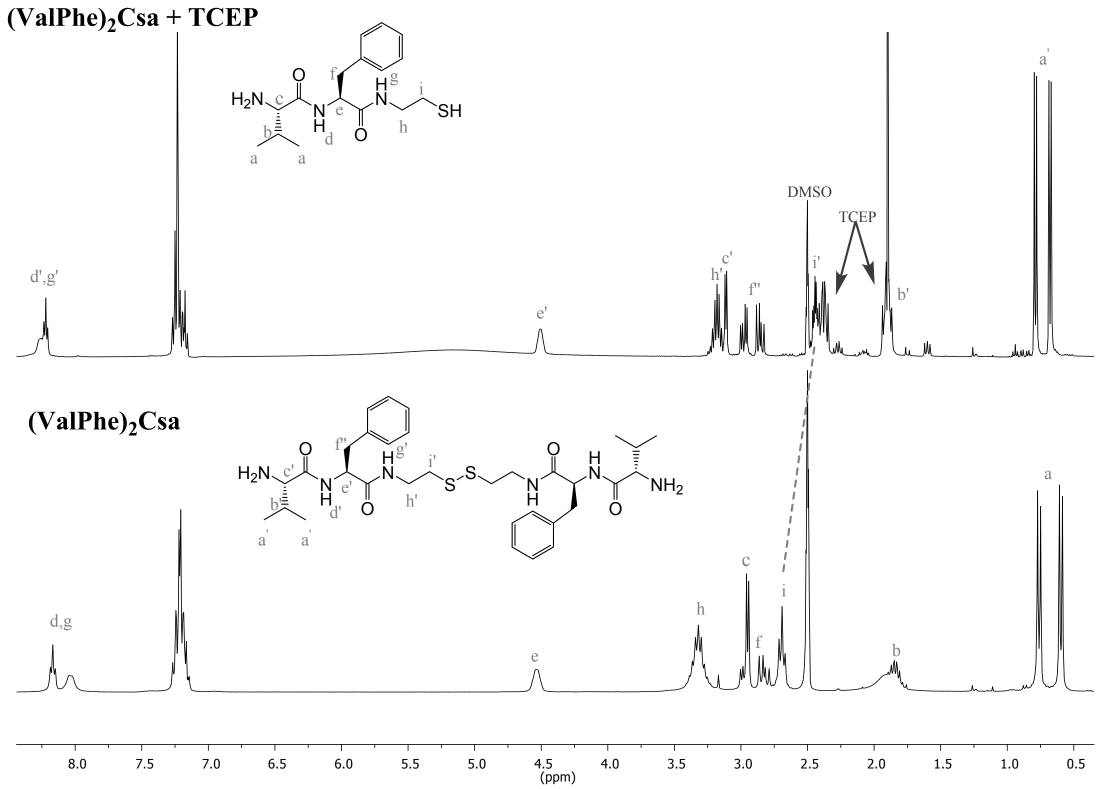

Figure S6. ^{1}H NMR spectra before and after treating with TCEP (12 mM) a sample of **(ValPhe)$_2$Csa** (9.3mM).

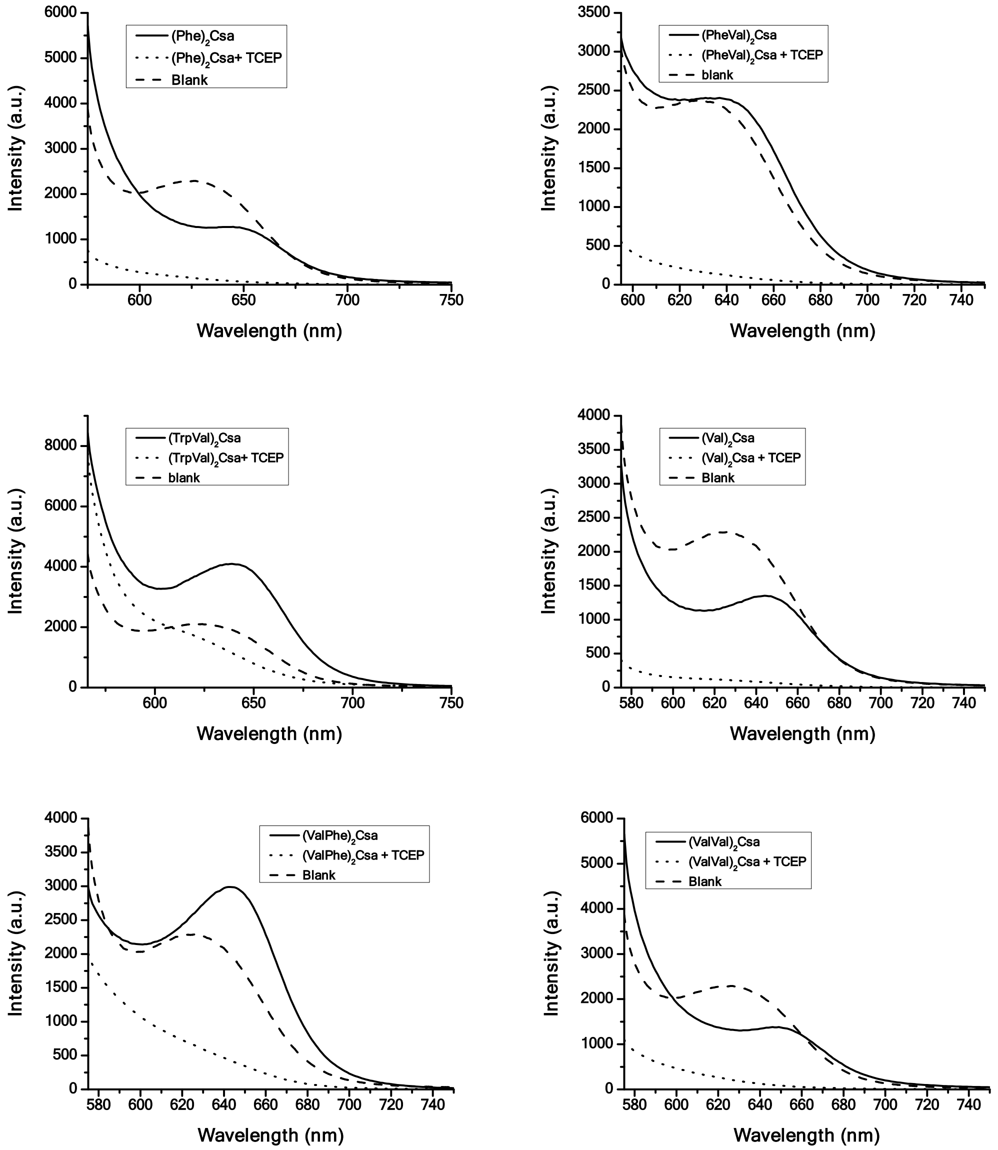

Figure S7. Fluorescence spectra for the study of Nile Red loading.

SYNTHESIS AND CHARACTERIZATION:

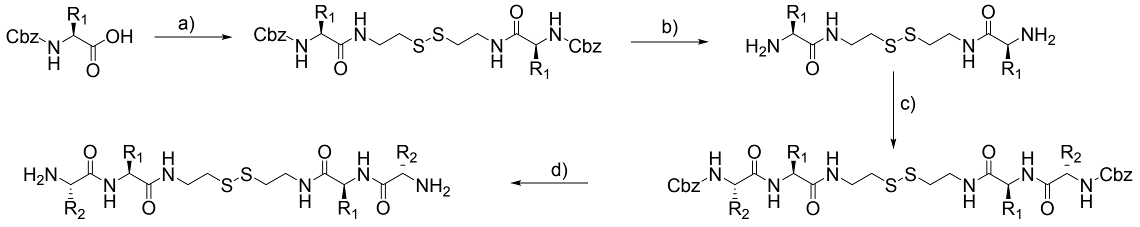

Scheme S1. *a) Cystamine dihydrochloride, DIPEA, TBTU, DMF, 16 h; b)HBr 33 wt.% in acetic acid, 1h; c) Cbz-L-Amino acid-OH, DIPEA, TBTU, DMF, 16 h; d) HBr 33 wt.% in acetic acid, 1h.*

Data for $(ZPhe)_2Csa$: dibenzyl ((2S,2'S)-((disulfanediylbis(ethane-2,1-diyl))bis(azanediyl))bis(1-oxo-3-phenylpropane-1,2-diyl))dicarbamate (Yield: 2.853 mmol, 64%): ^{1}H NMR (300 MHz, DMSO-d_6): δ 8.21 (t, *J* = 5.2 Hz, 1H), 7.49 (d, *J* = 8.5 Hz, 1H), 7.26 (m, 10H), 4.94 (m, 2H), 4.21 (td, *J* = 9.6, 4.7 Hz, 1H), 3.36 (m, 2H), 2.91 (ddd, 2H), 2.74 (m, 2H). ^{13}C NMR (75 MHz, DMSO-d_6): δ 171.5, 155.8, 138.0, 137.0, 129.1, 128.2, 128.0, 127.6, 127.4, 126.2, 65.2, 56.2, 38.0, 37.6, 36.9.

Data for $(ZPhePhe)_2Csa$: dibenzyl ((2S,5S,16S,19S)-5,16-dibenzyl-3,6,15,18-tetraoxo-1,20-diphenyl-10,11-dithia-4,7,14,17-tetraazaicosane-2,19-diyl)dicarbamate (Yield: 1.72 mmol, 85%): ^{1}H NMR (400 MHz, DMSO-d_6) δ 8.20 (m, 2H), 7.42 (d, *J* = 8.6 Hz, 1H), 7.24 (m, 15H), 4.91 (s, 2H), 4.52 (m, 1H), 4.26 (td, *J* = 10.2, 4.0 Hz, 1H), 3.34 (m, 2H), 2.90 (m, 6H). ^{13}C NMR (101 MHz, DMSO-d_6) δ 171.1, 170.7, 155.7, 137.9, 137.4, 136.9, 129.2, 129.1, 128.2, 128.0, 127.9, 127.9, 127.6, 127.3, 126.2, 126.1,126.1, 65.2, 56.0, 53.9, 38.0, 37.9, 37.4, 36.9. HR ESMS: m/z: calcd for $C_{56}H_{60}N_6O_8S_2$: 1009.3992; found: 1009.3995 [M + H+].

Data for $(ZPheVal)_2Csa$: dibenzyl ((2S,5S,16S,19S)-5,16-diisopropyl-3,6,15,18-tetraoxo-1,20-diphenyl-10,11-dithia-4,7,14,17-tetraazaicosane-2,19-diyl)dicarbamate (Yield: 6.704 mmol, 85 %): ^{1}H NMR (400 MHz, DMSO-d_6): δ 8.14 (t, *J* = 5.4 Hz, 1H, NH), 7.85 (d, *J* = 8.9 Hz, 1H, NH), 7.52 (d, *J* = 8.6 Hz, 1H, NH), 7.24 (m, 10H, CH-Ar), 4.93 (m, 2H, CH_2), 4.33 (td, *J* = 10.5, 4.1 Hz, 1H, CH), 4.14 (dd, *J* = 8.8, 6.9 Hz, 1H, CH), 3.34 (m, 2H, CH_2) 2.86 (m, 2H, CH_2), 2.77 (dd, *J* = 13.4, 6.8 Hz, 2H, CH_2), 1.93 (m, 1H, CH), 0.85 (m, 6H, CH_3). ^{13}C NMR (101 MHz, DMSO-d_6): δ 171.3 (C=O), 170.8 (C=O), 155.8 (C=O), 138.0 (C-Ar), 137.0 (C-Ar), 129.2 (CH-Ar), 128.2 (CH-Ar), 128.0, 127.6z 127.3, 126.2, 65.1, 57.6, 56.0, 37.8, 37.2, 37.0 , 30.8, 19.2, 18.1. HR ESMS: m/z: calcd for $C_{48}H_{60}N_6O_8S_2$: 913.3992; found: 913.4001 [M + H+].

Data for $(ZTrp)_2Csa$: dibenzyl((2S,2'S) -((disulfanediylbis(ethane-2,1-diyl))bis(azanediyl))bis(3-(1H-indol-3-yl)-1-oxopropane-1,2-diyl))dicarbamate (Yield: 7.0 mmol, 80%): ^{1}H NMR (400 MHz, DMSO-d_6): δ 10.79 (s, 1H), 8.16 (t, *J* = 5.0 Hz, 1H), 7.61 (d, *J* = 7.8 Hz, 1H), 7.30 (m, *J* = 19.5, 13.8, 7.2 Hz, 3H), 7.14 (s, 1H), 7.01 (dt, *J* = 36.5, 7.3 Hz, 1H), 4.95 (s, 1H), 4.25 (dd, *J* = 13.4, 8.5 Hz, 1H), 3.35 (m, 2H), 3.02 (m, *J* = 23.8, 14.5, 7.1 Hz, 2H), 2.71 (dd, *J* = 11.4, 4.7 Hz, 2H). ^{13}C NMR (101 MHz, DMSO-d_6): δ 171.8, 155.7, 136.9, 136.0, 128.2, 127.6, 127.4, 127.2, 123.7, 120.8, 118.4, 118.1, 111.2, 110.1, 65.2, 55.6, 38.1, 36.9, 27.9.

Data for $(ZTrpVal)_2Csa$: dibenzyl ((2S,5S,16S,19S)-1,20-di(1H-indol-3-yl)-5,16-diisopropyl-3,6,15,18-tetraoxo-10,11-dithia-4,7,14,17-tetraazaicosane-2,19-diyl)dicarbamate (Yield: 5.89 mmol, 87%): ^{1}H NMR (400 MHz, DMSO-d_6): δ 10.79 (s, 1H), 8.09 (t, *J* = 5.4 Hz, 1H), 7.80 (d, *J* = 8.8 Hz, 1H), 7.60 (d, *J* = 7.9 Hz, 1H), 7.43 (d, *J* = 8.4 Hz, 1H), 7.29 (m, *J* = 24.8, 15.8, 6.2 Hz, 6H), 7.15(m, 1H), 7.05 (t, *J* = 7.1 Hz, 1H), 6.96 (t, *J* = 7.4 Hz, 1H), 4.96 (m, 2H), 4.37 (td, *J* = 9.4, 4.6 Hz, 1H), 4.16 (dd, *J* = 8.8, 6.9 Hz, 1H), 3.30 (m, 2H), 3.02 (ddd, *J* = 24.5, 14.6, 6.9 Hz, 2H), 2.77 (t, *J* = 6.6 Hz, 2H), 1.97 (m, 1H), 0.84 (m, 6H). ^{13}C NMR (101 MHz, DMSO-d_6): δ 171.5, 170.8, 155.7, 136.9, 136.0, 128.2, 128.2, 127.7, 127.6, 127.3, 127.3, 123.7, 120.8, 118.4, 118.1, 111.2, 110.1, 65.2, 57.6, 55.5, 37.8, 37.0, 30.8, 27.6, 19.1, 18.0. HR ESMS: m/z: calcd for $C_{52}H_{62}N_8O_8S_2$: 991.4210; found: 991.4202 [M + H+].

Data for $(BocVal)_2Csa$: di-tert-butyl ((2S,2'S)-((disulfanediylbis(ethane-2,1-diyl))bis(azanediyl))bis(3-methyl-1-oxobutane-1,2-diyl))dicarbamate (Yield: 4.259 mmol, 80%). ^{1}H NMR (300 MHz, DMSO-d_6): δ 8.05 (t, *J* = 4.9 Hz, 1H), 6.60 (d, *J* = 8.9 Hz, 1H), 3.75 (t, *J* = 7.8 Hz, 1H), 3.35 (m, 2H), 2.78 (t, *J* = 6.6 Hz, 2H), 1.92

(dq, *J* = 13.2, 6.6 Hz, 1H), 1.38 (s, 9H), 0.83 (t, *J* = 6.2 Hz, 6H). ^{13}C NMR (75 MHz, DMSO-d$_6$): δ 171.5, 155.4, 77.95, 59.7, 37.8, 37.0, 30.3, 28.15, 19.2, 18.1.

Data for (BocValPhe)$_2$Csa: di-tert-butyl ((3S,6S,17S,20S)-6,17-dibenzyl-2,21-dimethyl-4,7,16,19-tetraoxo-11,12-dithia-5,8,15,18-tetraazadocosane-3,20-diyl)dicarbamate (Yield: 3.294 mmol, 88%): ^{1}H NMR (300 MHz, DMSO-d$_6$): δ 8.14 (s, 1H), 7.88 (d, *J* = 8.3 Hz, 1H), 7.19 (m, 5H), 6.66 (d, *J* = 8.8 Hz, 1H), 4.53 (dd, *J* = 14.0, 8.1 Hz, 1H), 3.72 (t, *J* = 7.8 Hz, 1H), 3.32 (s, 2H), 2.88 (ddd, *J* = 22.5, 13.7, 7.2 Hz, 2H), 2.66 (m, 2H), 1.82 (m, 1H), 1.37 (s, *J* = 21.3 Hz, 9H), 0.70 (dd, *J* = 13.8, 6.7 Hz, 6H). ^{13}C NMR (75 MHz, DMSO-d$_6$): δ 170.9, 170.9, 155.4, 137.5, 129.1, 128.0, 126.2, 78.1, 60.0, 53.6, 37.9, 36.9, 30.4, 28.1, 19.0, 18.1.

Data for (ZValTrp)$_2$Csa: dibenzyl ((3S,6S,17S,20S)-6,17-bis((1H-indol-3-yl)methyl)-2,21-dimethyl-4,7,16,19-tetraoxo-11,12-dithia-5,8,15,18-tetraazadocosane-3,20-diyl)dicarbamate (Yield: 6.34 mmol, 92 %): ^{1}H NMR (400 MHz, DMSO) δ 10.76 (d, *J* = 9.7 Hz, 1H), 8.04 (dd, *J* = 12.3, 6.8 Hz, 1H), 7.95 (d, *J* = 8.0 Hz, 1H), 7.55 (d, *J* = 7.7 Hz, 1H), 7.31 (m, 6H), 7.12 (s, 1H), 7.03 (dt, *J* = 13.6, 3.3 Hz, 1H), 6.94 (dt, *J* = 18.4, 5.7 Hz, 1H), 5.01 (m, 2H), 4.53 (m, 1H), 3.87 (m, 1H), 3.02 (ddd, *J* = 22.1, 14.6, 7.0 Hz, 2H), 2.81 (s, 2H), 2.59 (s, 2H), 1.92 (dd, *J* = 13.4, 6.5 Hz, 1H), 0.76 (d, *J* = 6.8 Hz, 6H). ^{13}C NMR (101 MHz, DMSO) δ 171.3, 170.9, 156.2, 137.0, 136.0, 128.4, 128.3, 127.8, 127.8, 127.6, 127.3, 123.6, 120.8, 118.4, 118.2, 111.2, 109.8, 65.5, 58.1, 53.4, 38.0, 36.8, 30.4, 28.0, 19.1, 18.0. HR ESMS: m/z: calcd for $C_{52}H_{62}N_8O_8S_2$: 991.4210; found: 991.4207 [M + H+].

Data for (ZValVal)$_2$Csa: dibenzyl ((3S,6S,17S,20S)-6,17-diisopropyl-2,21-dimethyl-4,7,16,19-tetraoxo-11,12-dithia-5,8,15,18-tetraazadocosane-3,20-diyl)dicarbamate (Yield: 3.32 mmol, 82 %): ^{1}H NMR (400 MHz, DMSO-d$_6$): δ 8.15 (t, *J* = 5.4 Hz, 1H), 7.69 (d, *J* = 8.8 Hz, 1H), 7.32 (m, 7H), 5.04 (s, 2H), 4.13 (dd, *J* = 8.7, 7.2 Hz, 1H), 3.92 (dd, *J* = 8.9, 7.0 Hz, 1H), 3.30 (m, 2H), 2.76 (t, *J* = 6.6 Hz, 2H), 1.94 (m, *J* = 19.1, 13.6, 6.7 Hz, 2H), 0.84 (m, 12H).^{13}C NMR (101 MHz, DMSO-d$_6$): δ 170.9, 170.8, 156.0, 137.0, 128.2, 127.6, 127.5, 65.3, 60.29, 57.5, 37.7, 37.0, 30.6, 30.1, 19.2, 19.1, 18.2, 18.1. HR ESMS: m/z: calcd for $C_{40}H_{60}N_6O_8S_2$: 817.3994.; found: 817.3992[M + H+].

NMR SPECTRA:

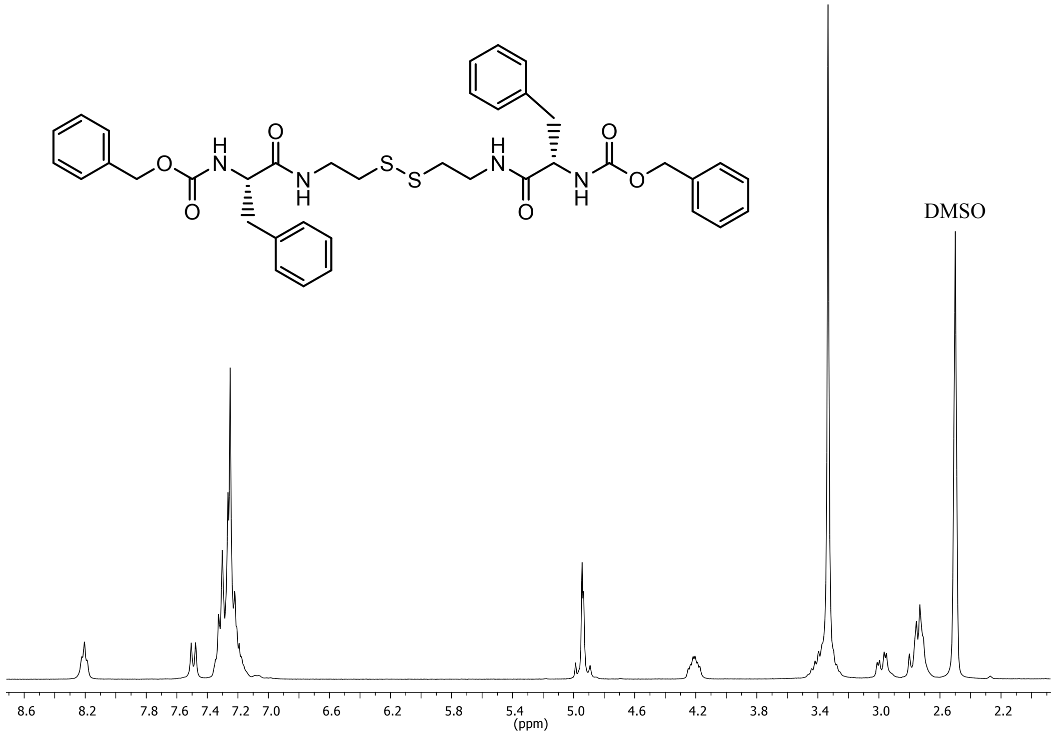

Figure S8. *^{1}H NMR spectrum of compound **(ZPhe)$_2$Csa**.*

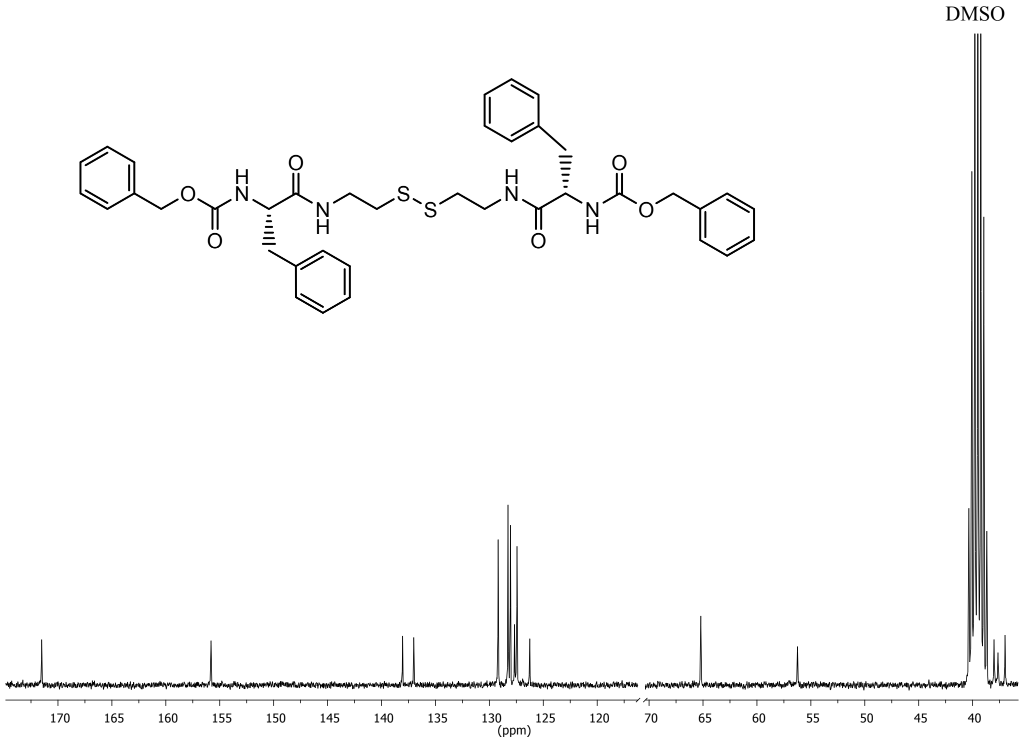

Figure S9*. ^{13}C NMR spectrum of compound* ***(ZPhe)$_2$Csa.***

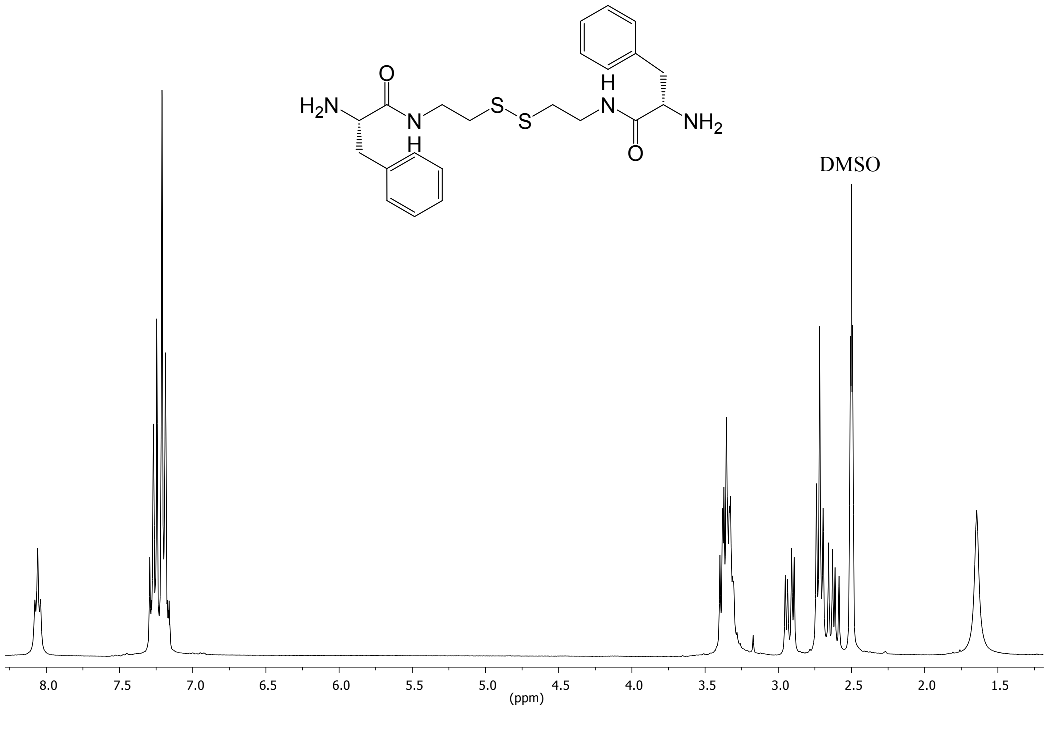

Figure S10*. ^{1}H NMR spectrum of compound* ***(Phe)$_2$Csa.***

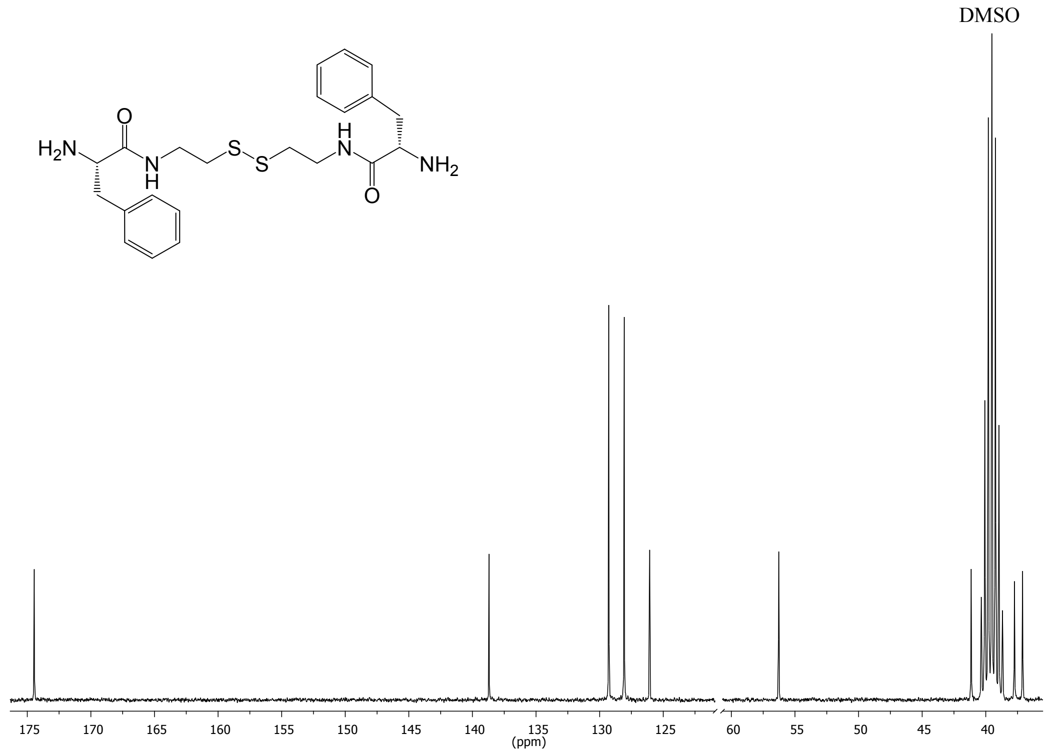

Figure S11. *^{13}C NMR spectrum of compound* ***(Phe)$_2$Csa.***

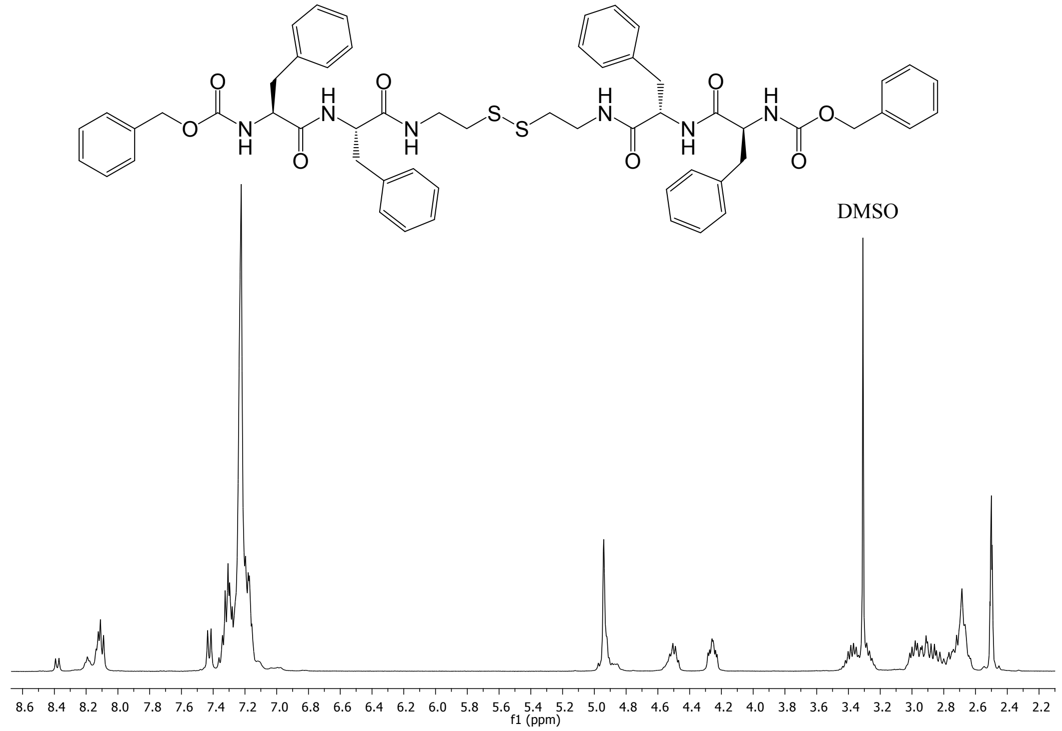

Figure S12. *^{1}H NMR spectrum of compound* ***(ZPhePhe)$_2$Csa***.

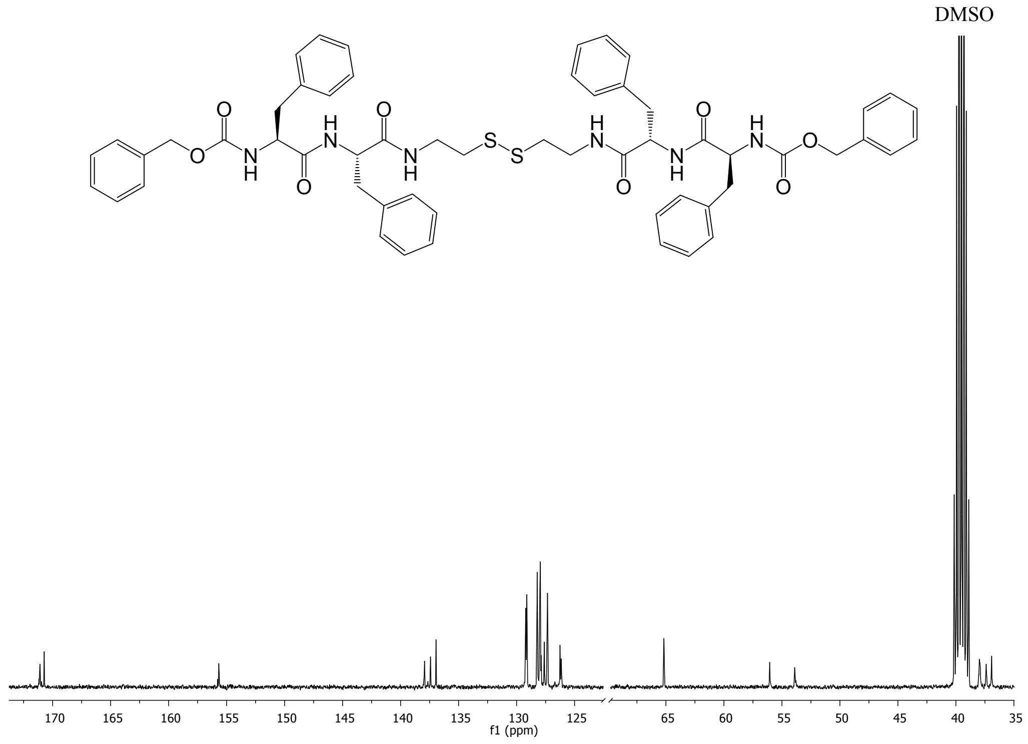

Figure S13. *^{13}C NMR spectrum of compound* ***(ZPhePhe)$_2$Csa****.*

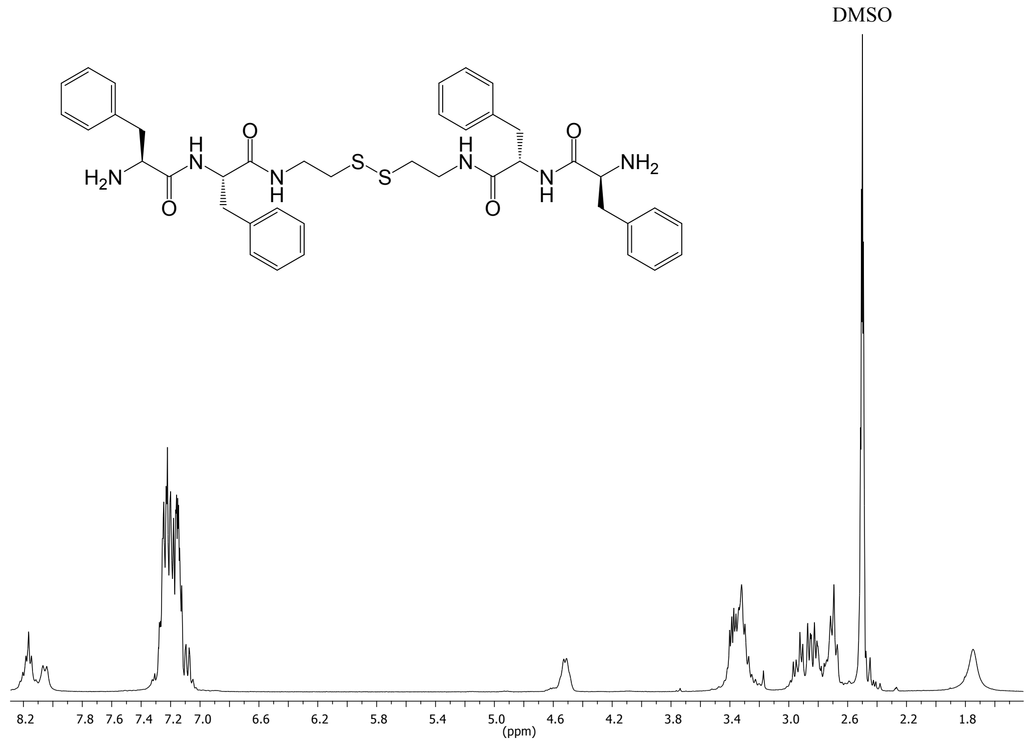

Figure S14. *^{1}H NMR spectrum of compound* ***(PhePhe)$_2$Csa****.*

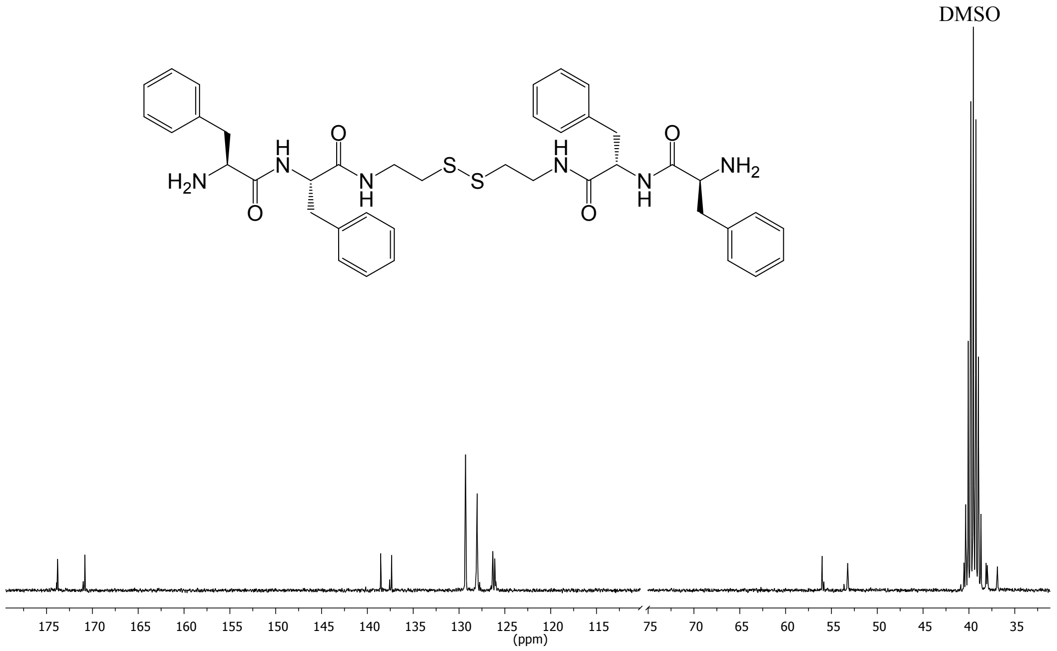

Figure S15. *^{13}C NMR spectrum of compound (**PhePhe)₂Csa**.*

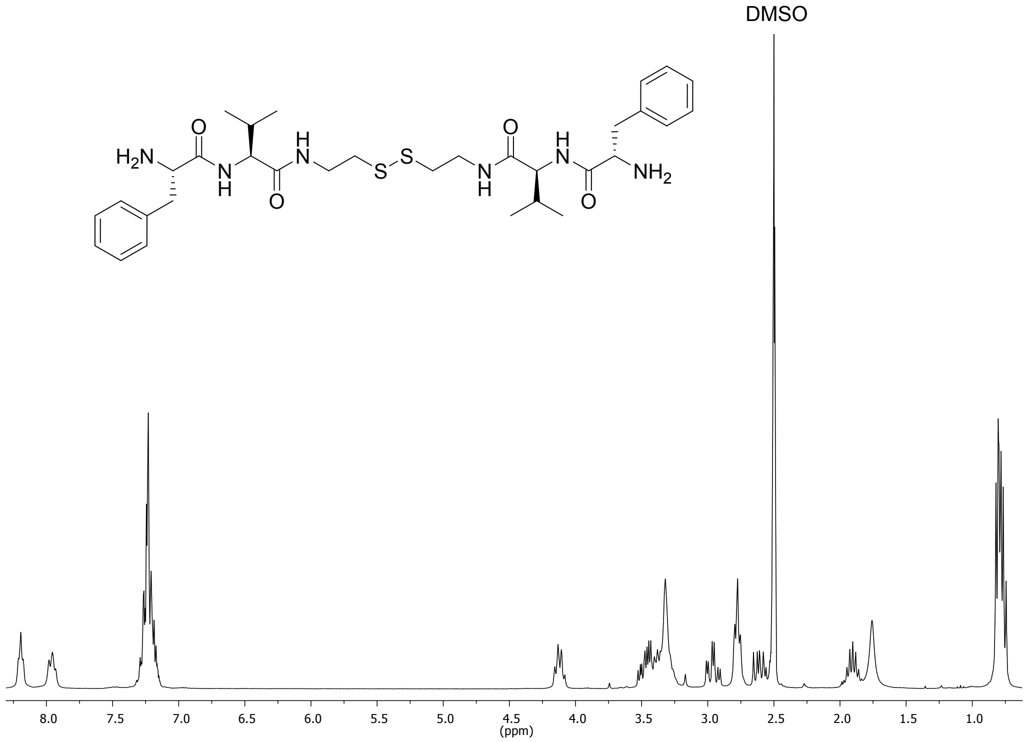

Figure S16. *^{1}H NMR spectrum of compound (**PheVal)₂Csa**.*

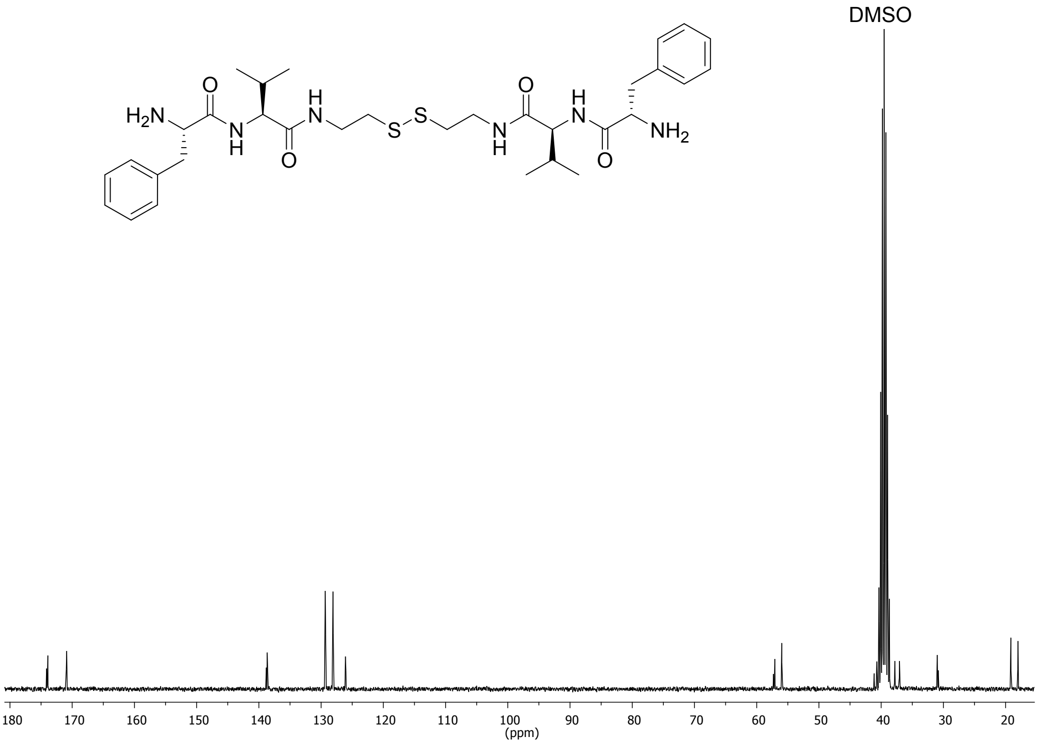

Figure S3. *^{13}C NMR spectrum of compound **(PheVal)$_2$Csa**.*

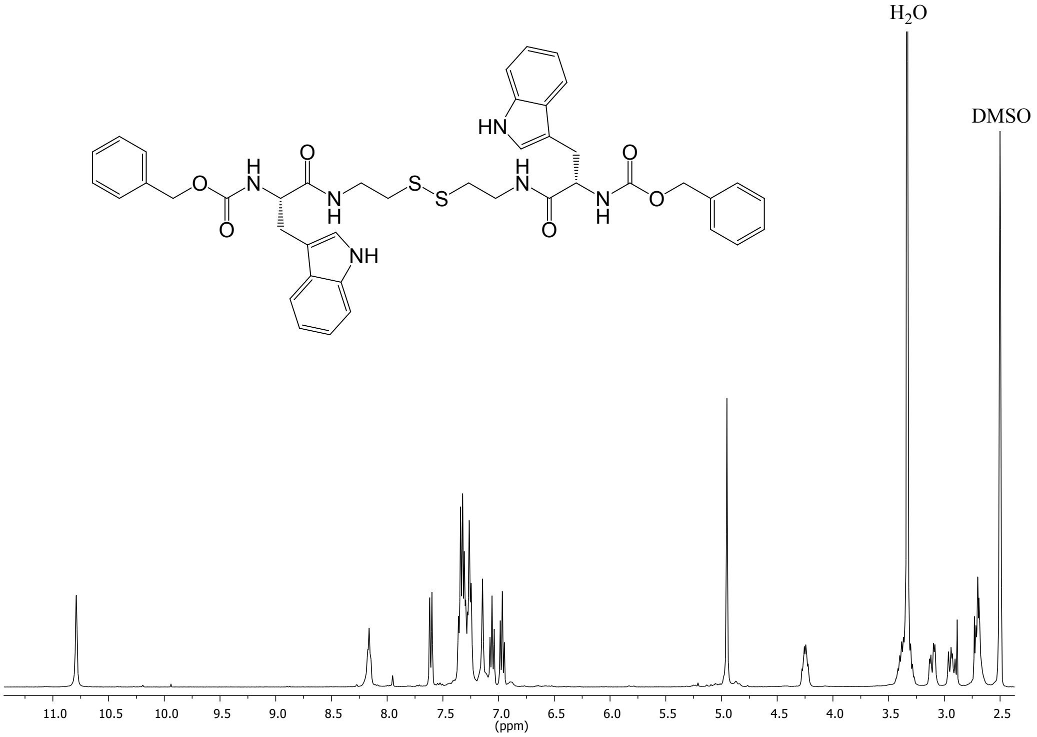

Figure S18. *^{13}H NMR spectrum of compound **(ZTrp)$_2$Csa**.*

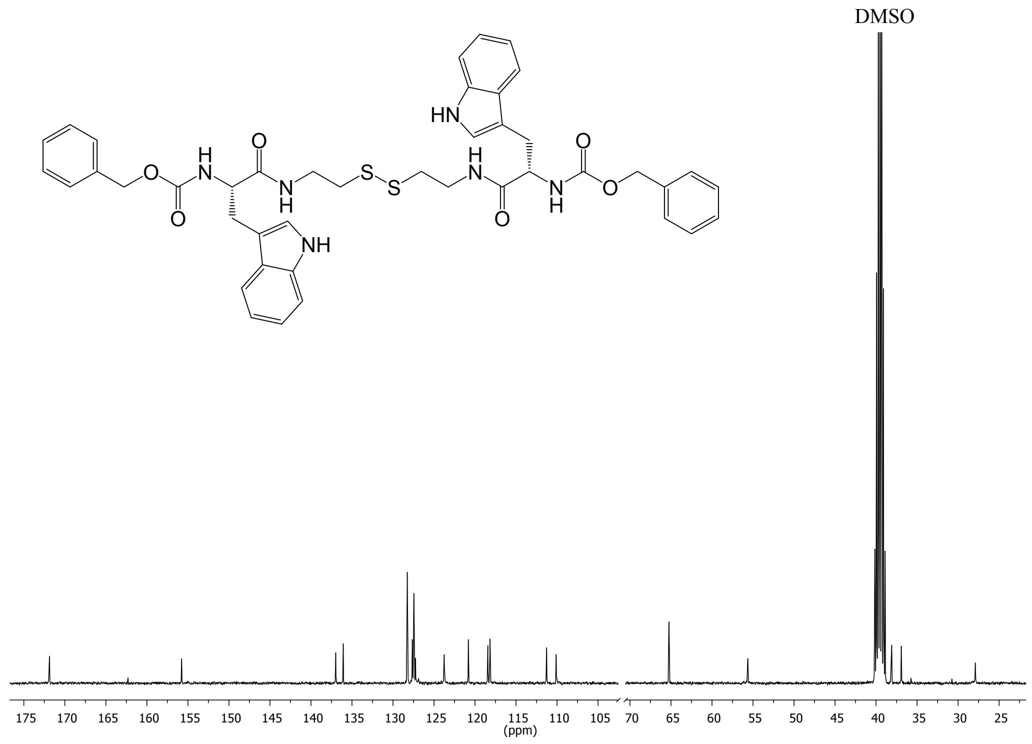

Figure S19. *^{13}C NMR spectrum of compound **(ZTrp)$_2$Csa**.*

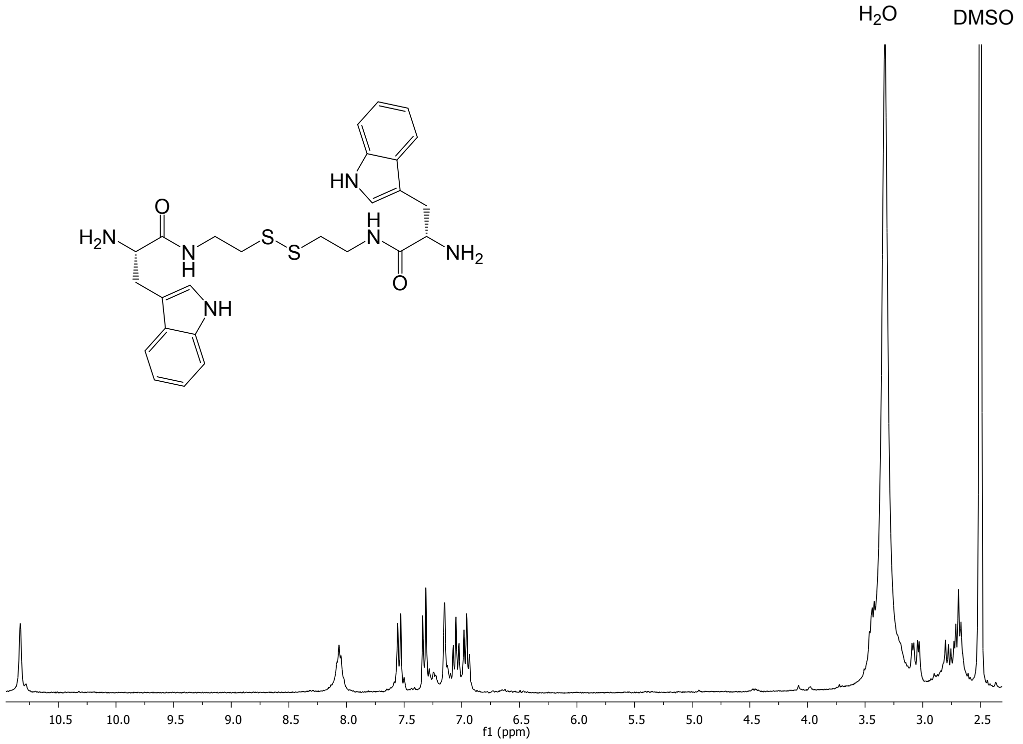

Figure S20. *^{1}H NMR spectrum of compound **(Trp)$_2$Csa**.*

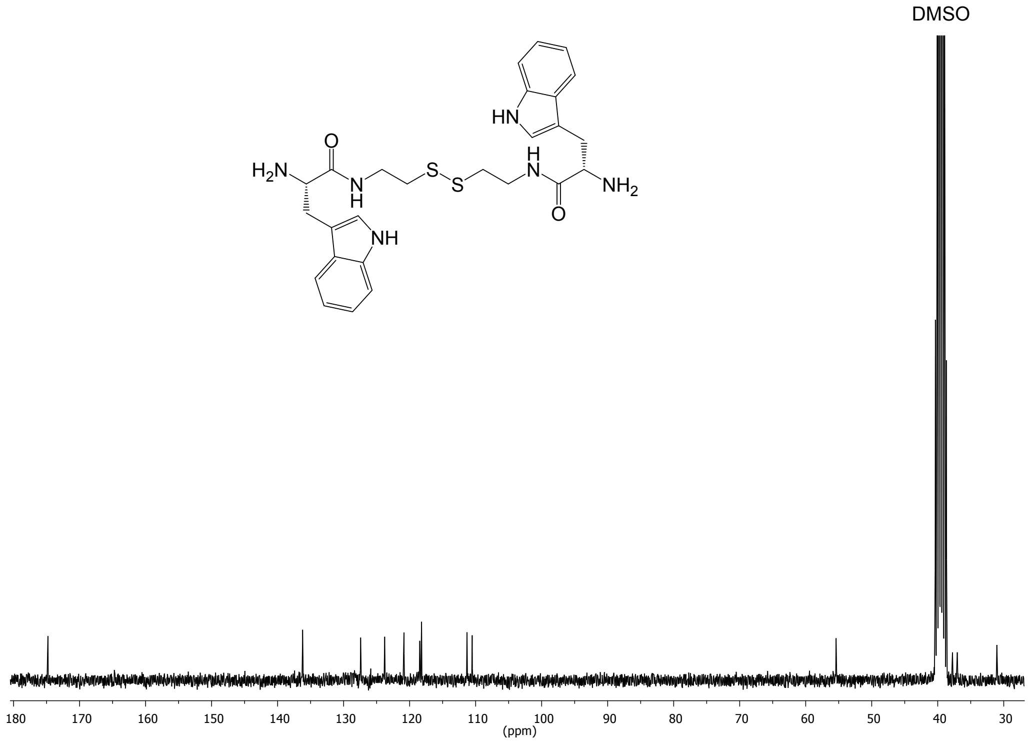

Figure S21 *^{13}C NMR spectrum of compound **$(Trp)_2Csa$**.*

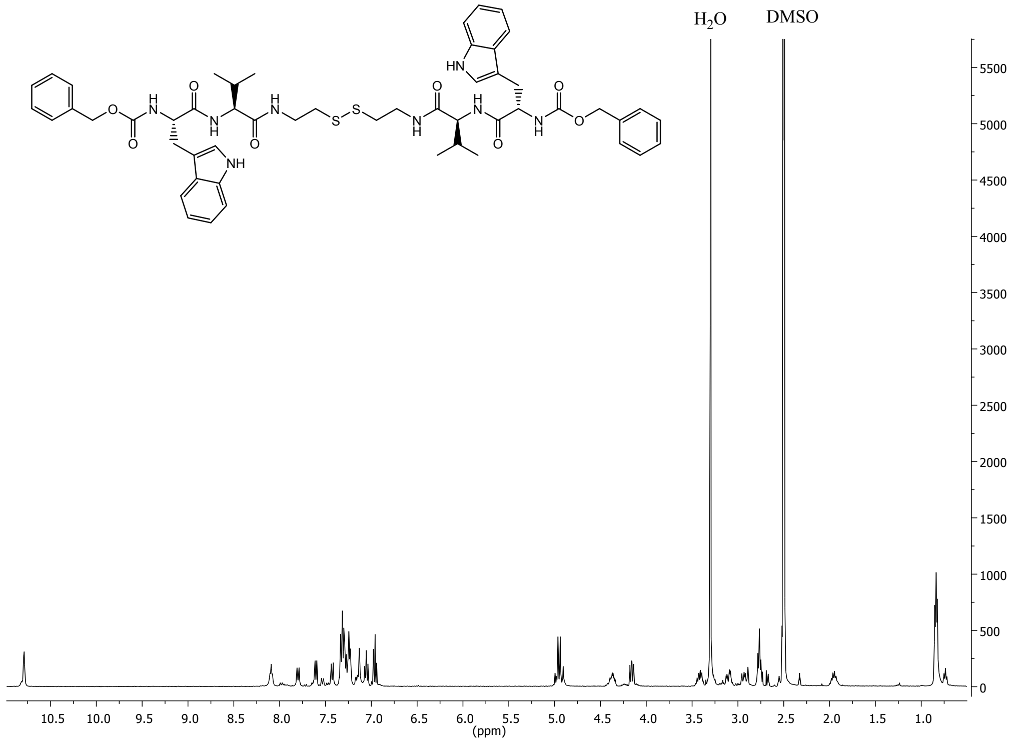

Figure S22. *^{1}H NMR spectrum of compound **$(ZTrpVal)_2Csa$**.*

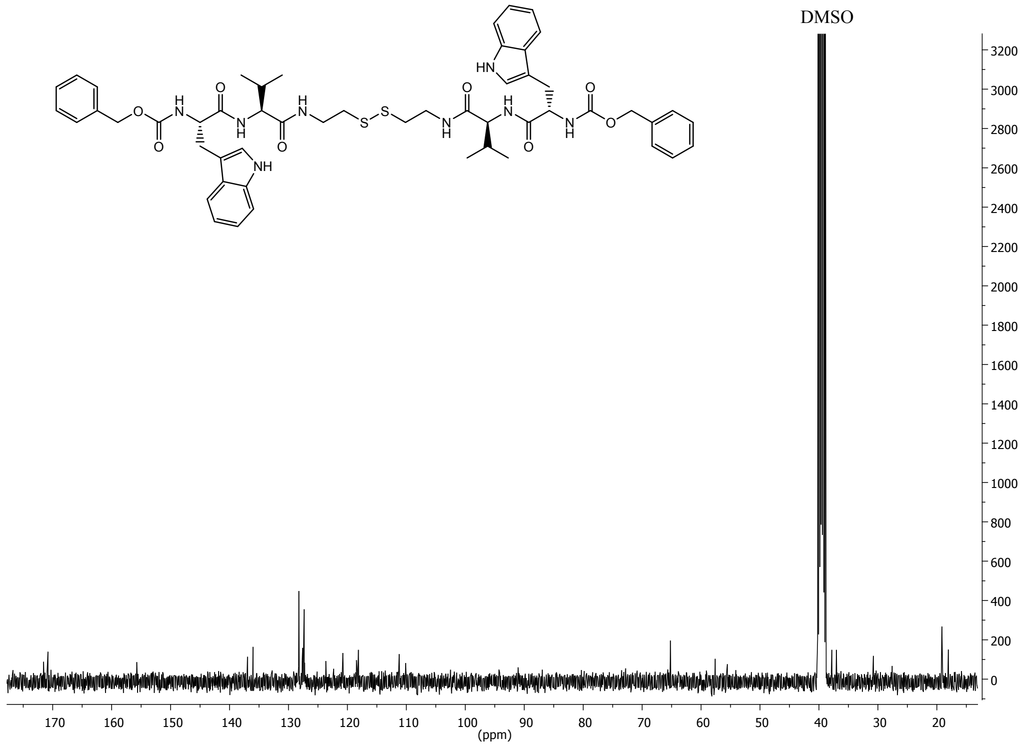

Figure S23. *^{13}C NMR spectrum of compound* ***(TrpVal)$_2$Csa****.*

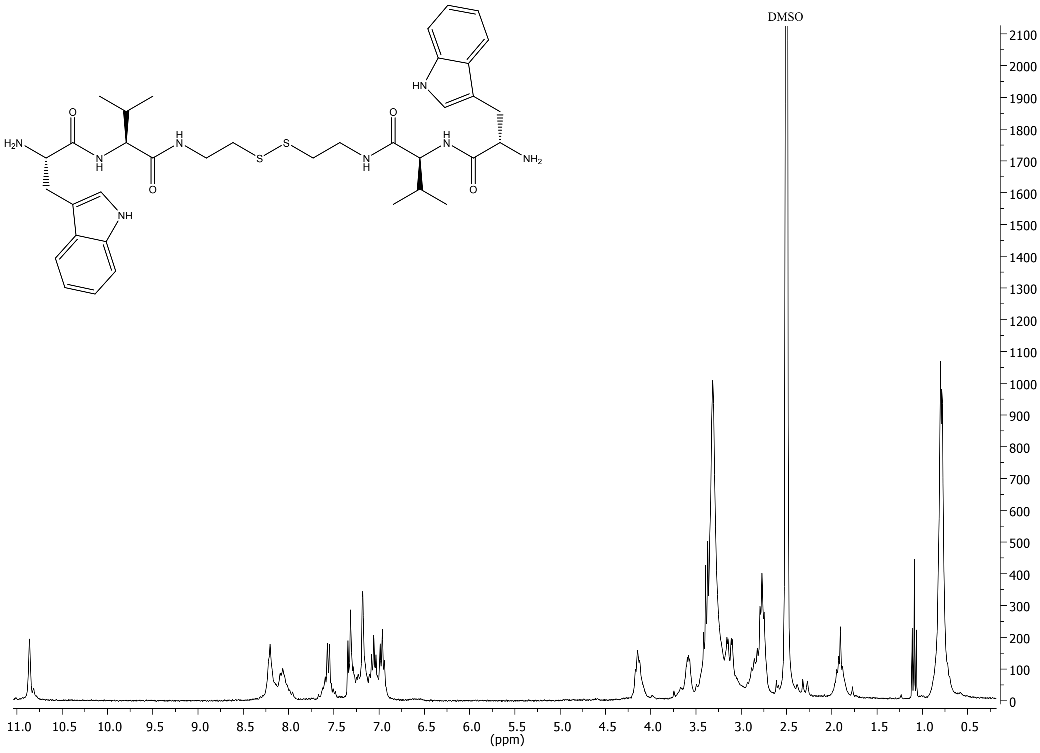

Figure S24. *^{1}H NMR spectrum of compound (****TrpVal)$_2$Csa****.*

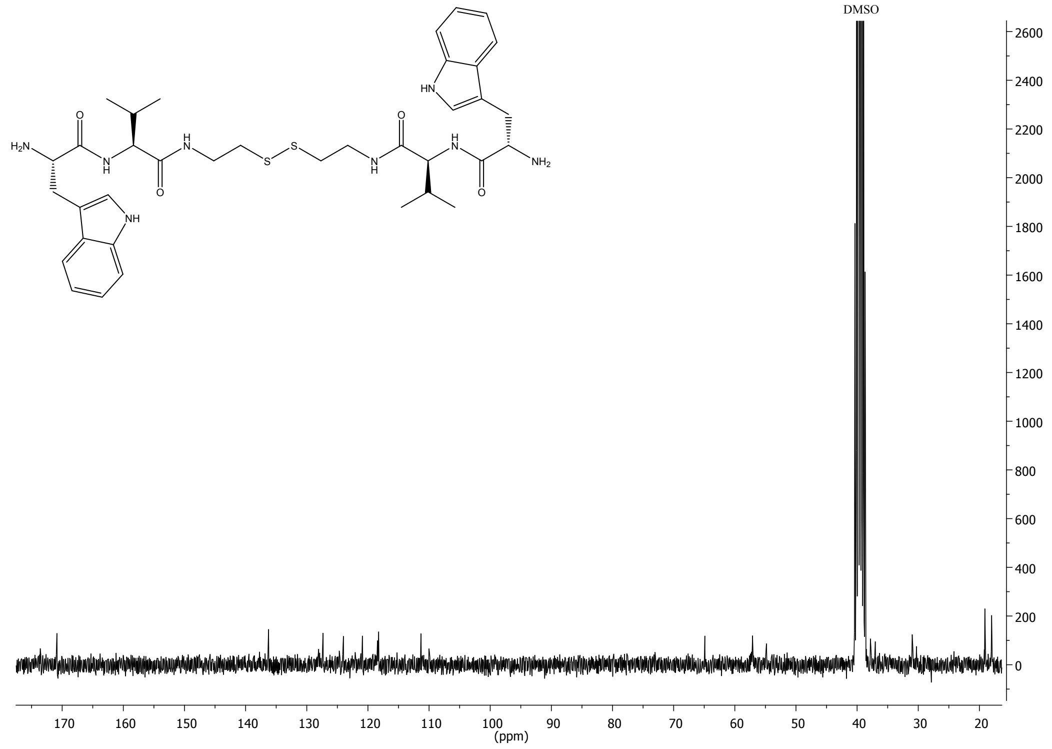

Figure S25. *^{13}C NMR spectrum of compound **(TrpVal)$_2$Csa**.*

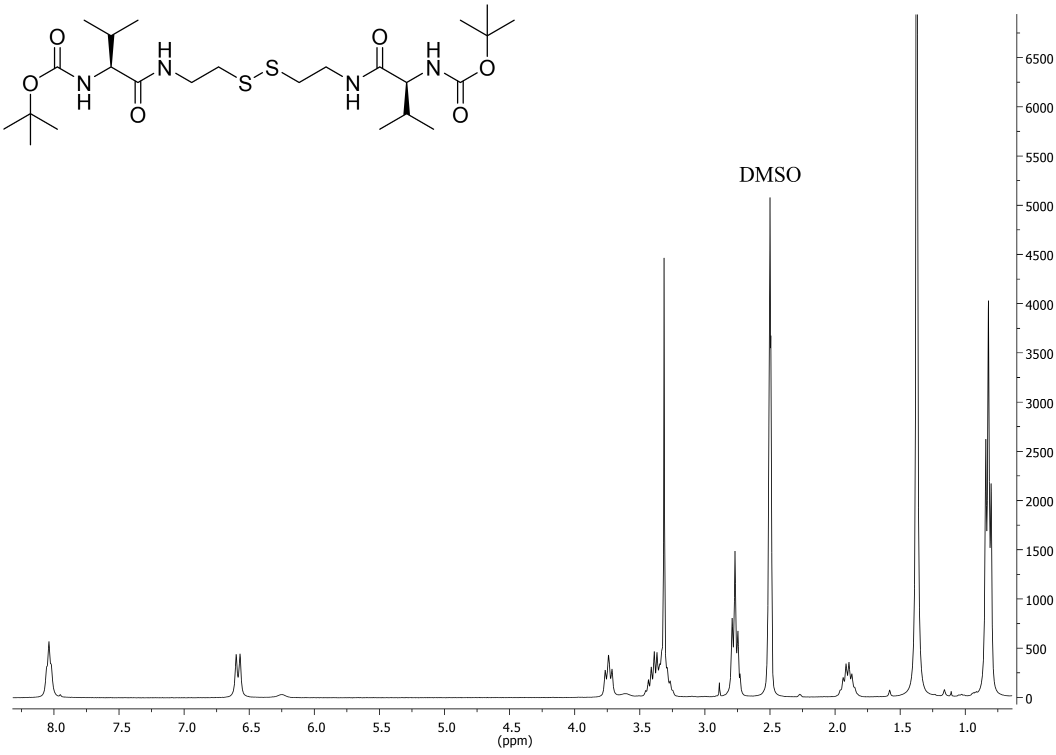

Figure S26. *^{1}H NMR spectrum of compound **(BocVal)$_2$Csa**.*

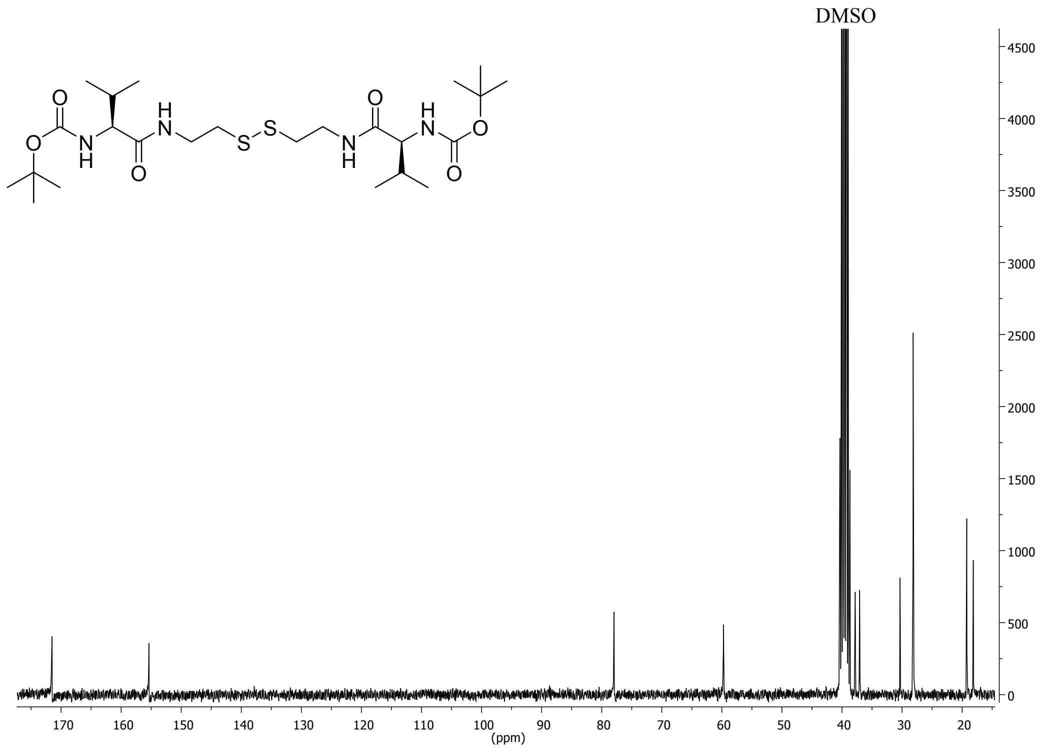

Figure S27. *^{13}C NMR spectrum of compound* ***(BocVal)$_2$Csa****.*

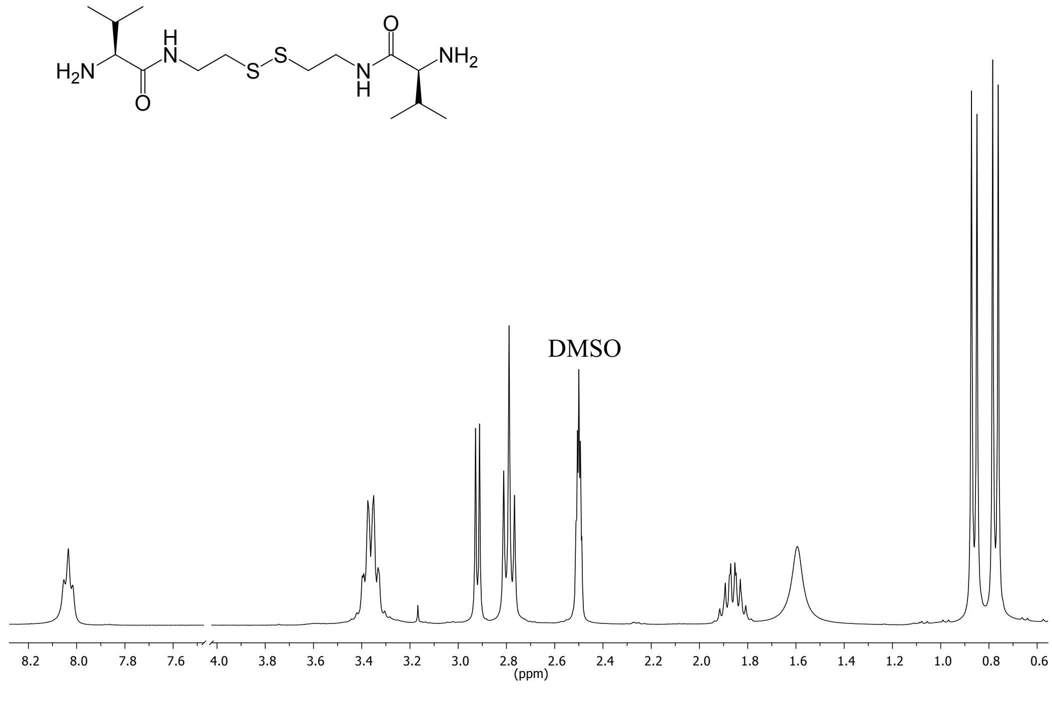

Figure S28. *^{1}H NMR spectrum of compound* ***(Val)$_2$Csa****.*

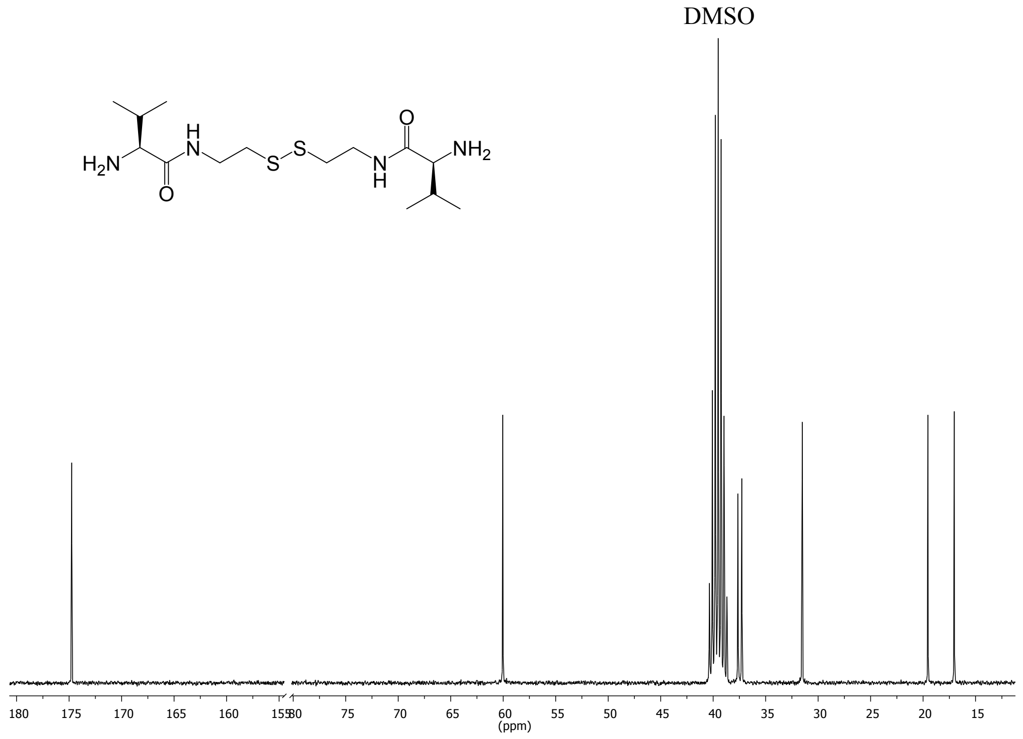

Figure S29. *^{13}C NMR spectrum of compound* ***(Val)$_2$Csa***.

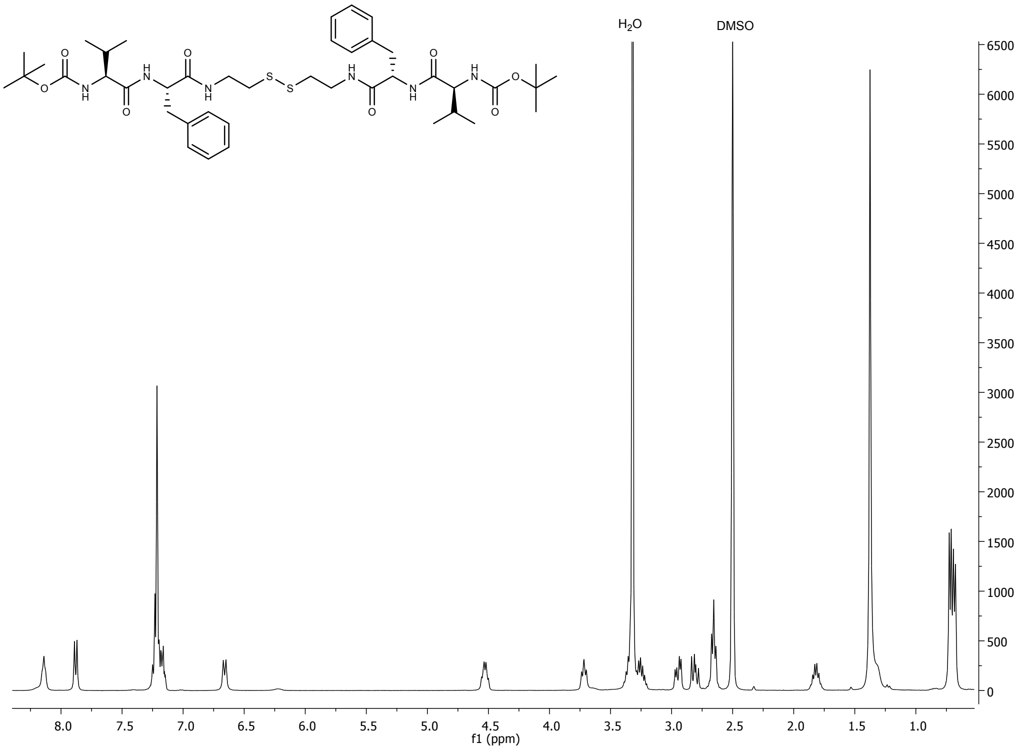

Figure S30. *^{1}H NMR spectrum of compound* ***(BocValPhe)$_2$Csa***.

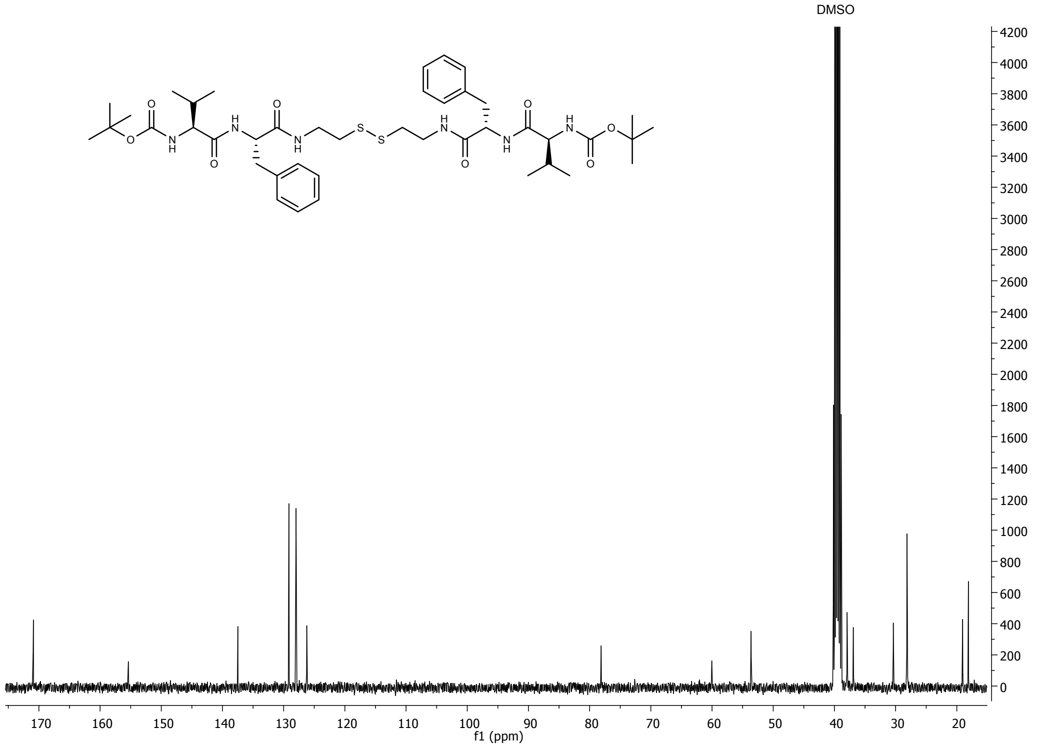

Figure S31.[13]*C NMR spectrum of compound* ***(BocValPhe)₂Csa***.

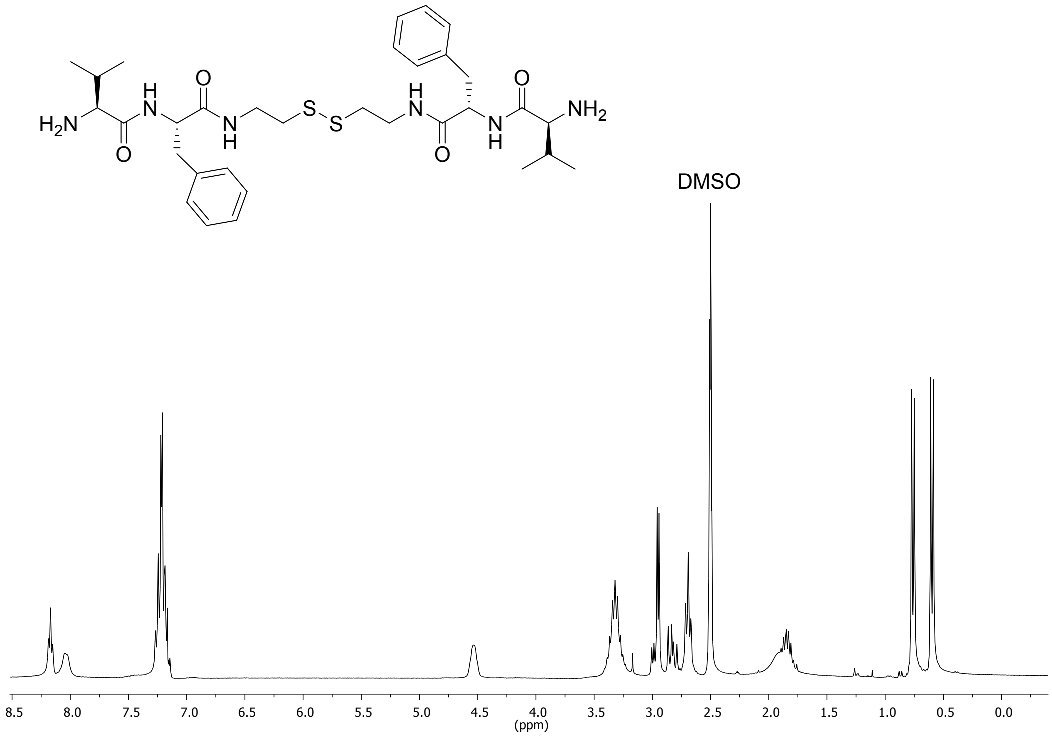

Figure S32. [1]*H NMR spectrum of compound* ***(ValPhe)₂Csa***.

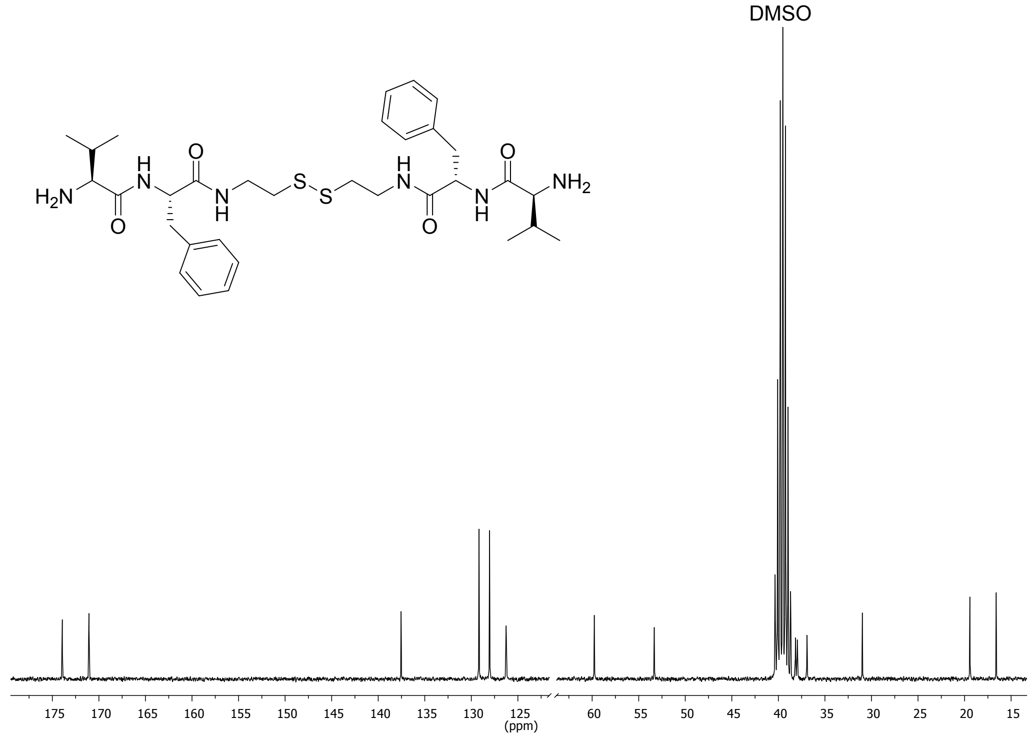

Figure S33. ^{13}C *NMR spectrum of compound* ***(ValPhe)$_2$Csa***.

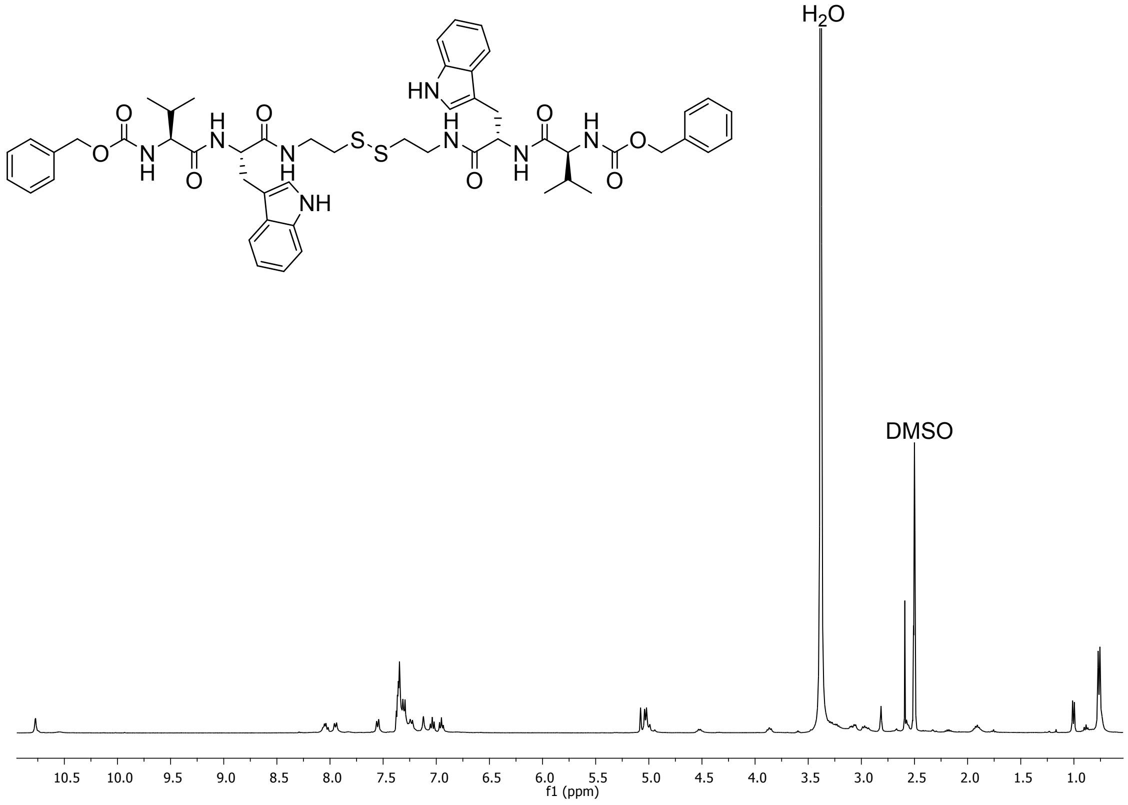

Figure S34. ^{1}H *NMR spectrum of compound* ***(ZValTrp)$_2$Csa***.

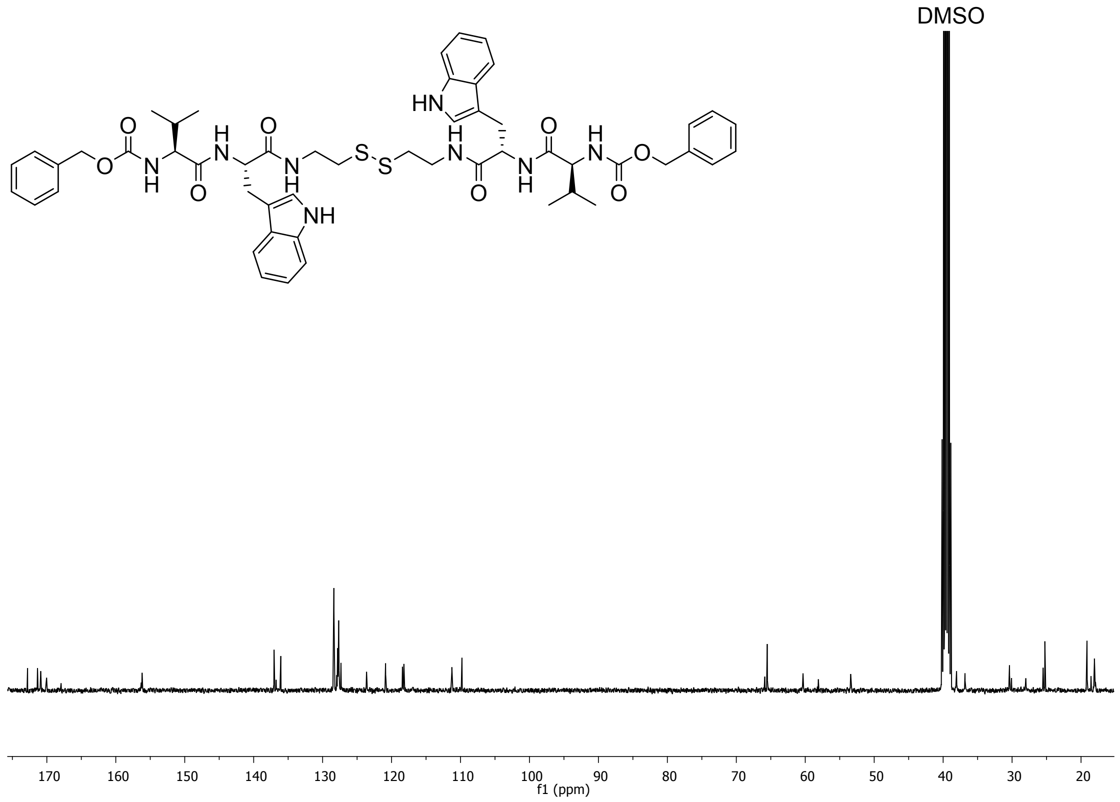

Figure S35. *^{13}C NMR spectrum of compound* ***(ZValTrp)$_2$Csa****.*

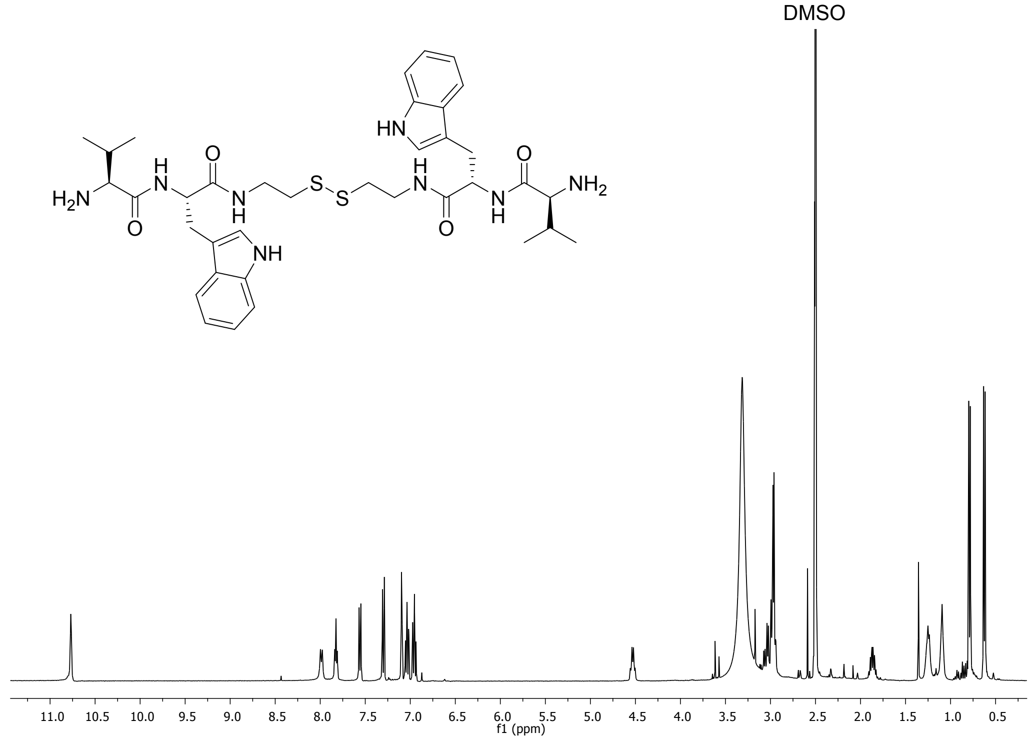

Figure S36. *^{1}H NMR spectrum of compound* ***(ValTrp)$_2$Csa****.*

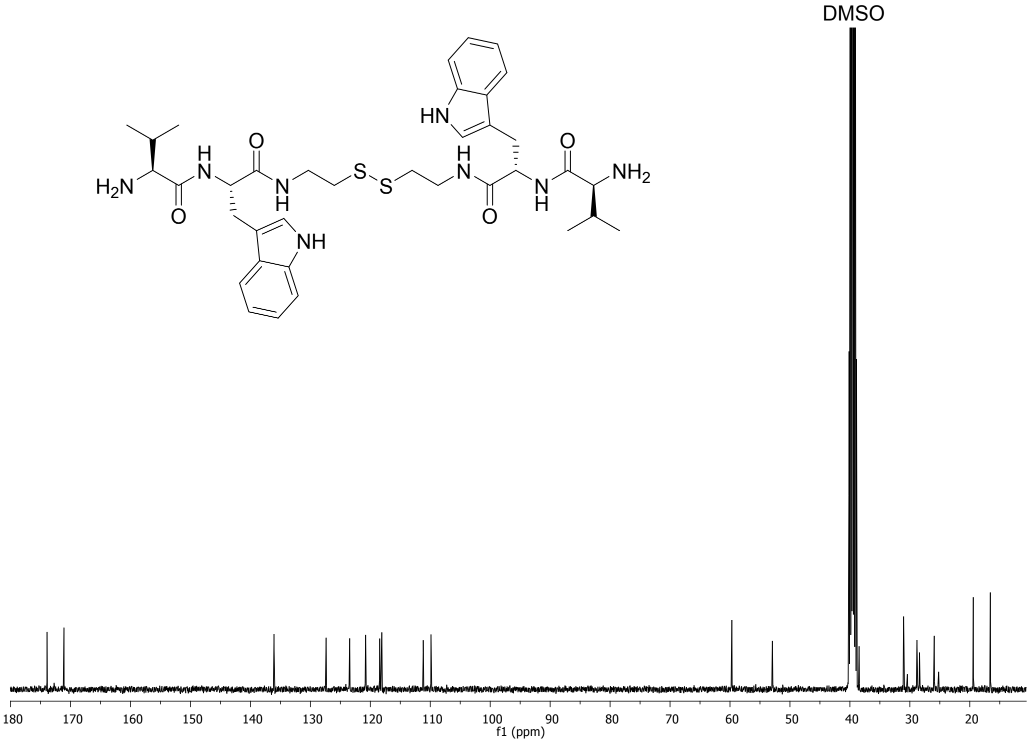

Figure S37. *^{13}C NMR spectrum of compound* ***(ValTrp)$_2$Csa****.*

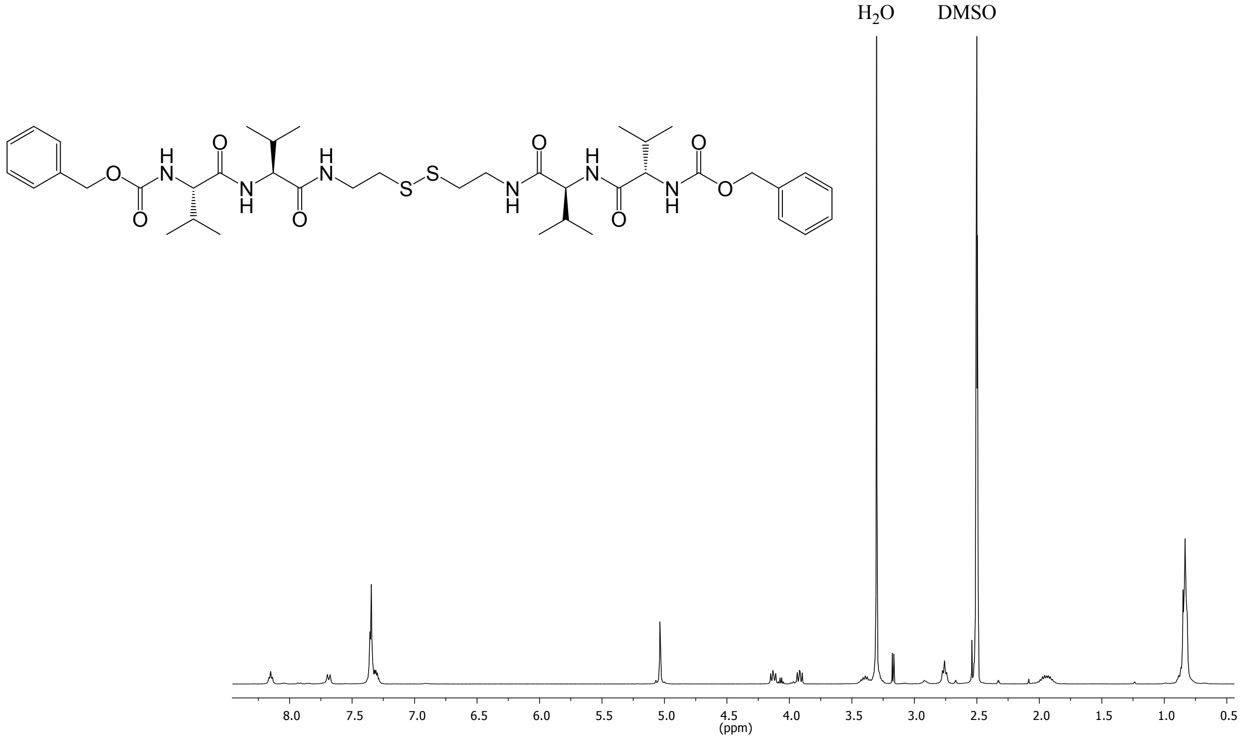

Figure S38. *^{1}H NMR spectrum of compound* ***(ZValVal)$_2$Csa****.*

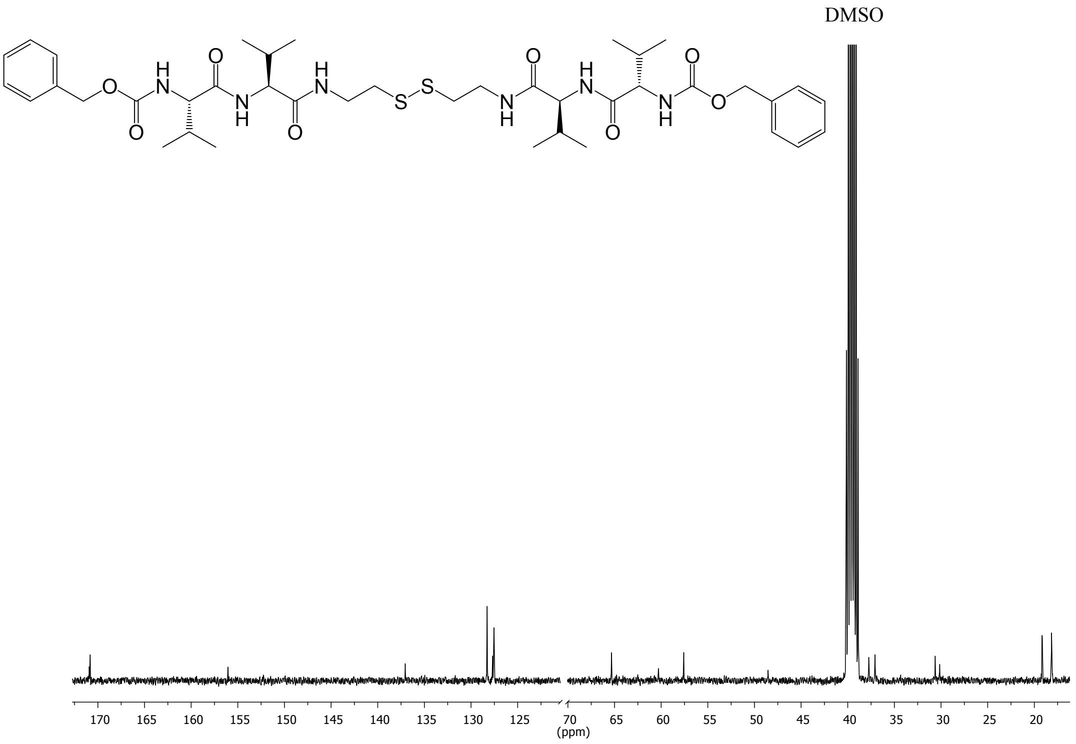

Figure S39. *[31]C NMR spectrum of compound* ***(ZValVal)₂Csa***.

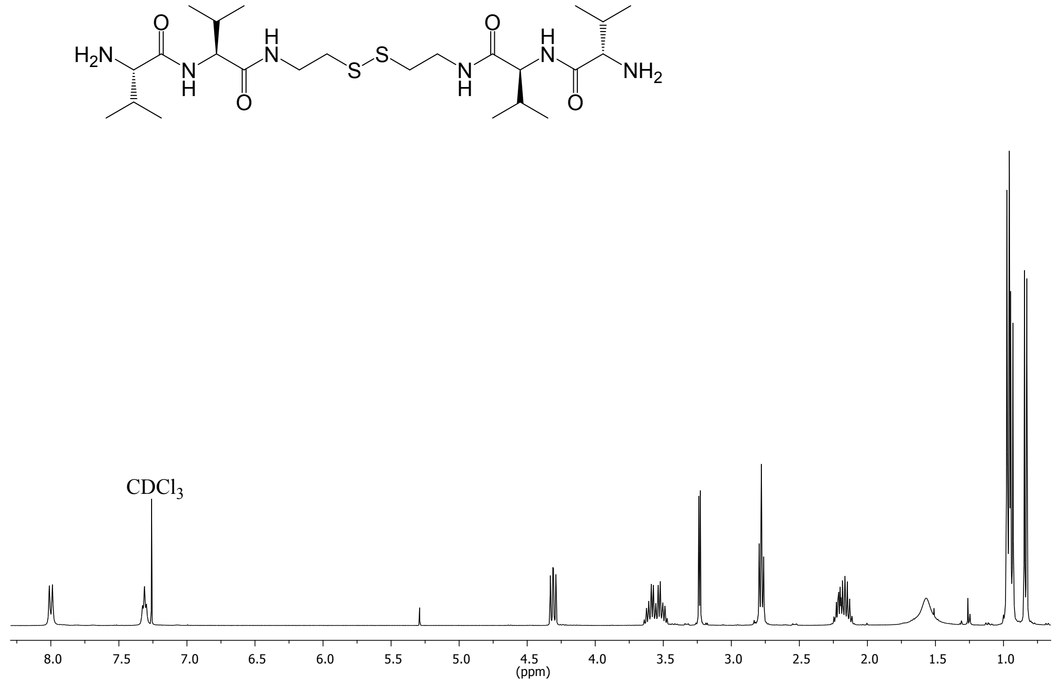

Figure S40. *^{1}H NMR spectrum of compound* ***(ValVal)₂Csa.***

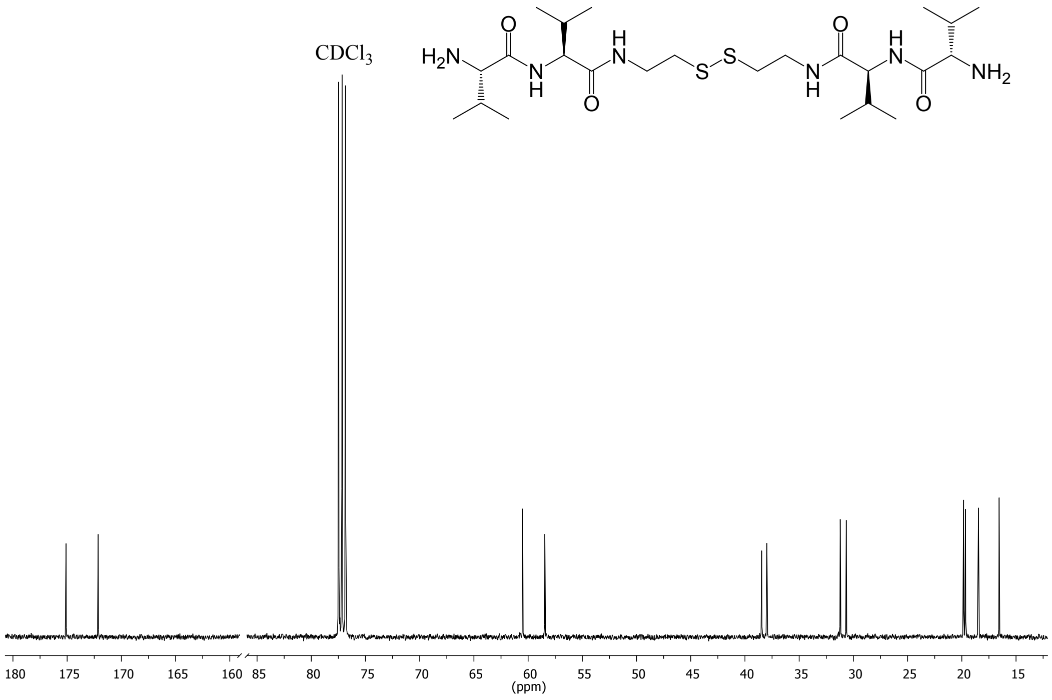

Figure S41 *^{13}C NMR spectrum of compound (**ValVal)$_2$Csa**.*
